# PET imaging of brain aromatase in humans and rhesus monkeys by ^11^C-labeled cetrozole analogs

**DOI:** 10.1038/s41598-021-03063-8

**Published:** 2021-12-08

**Authors:** Kayo Takahashi, Takamitsu Hosoya, Kayo Onoe, Tomoko Mori, Shusaku Tazawa, Aya Mawatari, Yasuhiro Wada, Yumiko Watanabe, Hisashi Doi, Yasuyoshi Watanabe

**Affiliations:** 1 RIKEN Center for Biosystems Dynamics Research, and Center for Life Science Technologies, 6-7-3 Minatojima-minamimachi, Chuo-ku, Kobe, Hyogo 650-0047 Japan; 2 Institute of Biomaterials and Bioengineering, Tokyo Medical and Dental University (TMDU), 2-3-10 Kanda-Surugadai, Chiyoda-ku, Tokyo, 101-0062 Japan

**Keywords:** Molecular neuroscience, Translational research

## Abstract

Aromatase is an estrogen synthetic enzyme that plays important roles in brain functions. To quantify aromatase expression in the brain by positron emission tomography (PET), we had previously developed [^11^C]cetrozole, which showed high specificity and affinity. To develop more efficient PET tracer(s) for aromatase imaging, we synthesized three analogs of cetrozole. We synthesized meta-cetrozole, nitro-cetrozole, and iso-cetrozole, and prepared the corresponding ^11^C-labeled tracers. The inhibitory activities of these three analogs toward aromatase were evaluated using marmoset placenta, and PET imaging of brain aromatase was performed using the ^11^C-labeled tracers in monkeys. The most promising analog in the monkey study, iso-cetrozole, was evaluated in the human PET study. The highest to lowest inhibitory activity of the analogs toward aromatase in the microsomal fraction from marmoset placenta was in the following order: iso-cetrozole, nitro-cetrozole, cetrozole, and meta-cetrozole. This order showed good agreement with the order of the binding potential (BP) of each ^11^C-labeled analog to aromatase in the rhesus monkey brain. A human PET study using [^11^C]iso-analog showed a similar distribution pattern of binding as that of [^11^C]cetrozole. The time–activity curves showed that elimination of [^11^C]iso-cetrozole from brain tissue was faster than that of ^11^C-cetrozole, indicating more rapid metabolism of [^11^C]iso-cetrozole. [^11^C]Cetrozole has preferable metabolic stability for brain aromatase imaging in humans, although [^11^C]iso-cetrozole might also be useful to measure aromatase level in living human brain because of its high binding potential.

## Introduction

Estrogen is involved in anxiety, depression, and Alzheimer’s disease^1,2^ and its substrate androgen is also related to depression and anxiety-like disorders^3,4^. Positron emission tomography (PET) is one of the most suitable techniques for investigating the dynamics of these hormones’ behavior, including their receptor systems in the body^5^. Several PET tracers for sex hormone systems have been developed. 16α-[^18^F]Fluoro-17β-estradiol is one of the most frequently used PET tracers for estrogen receptor imaging^6–8^, while [^18^F]fluorodihydrotestosterone has been used for androgen receptor imaging in animals^9,10^ and human studies^11,12^. These tracers have also been used for cancer and tumor imaging in clinical studies.

Estrogen is produced by aromatase, which catalyzes the demethylation of the androgen’s carbon 19. Aromatase is also involved in several brain functions such as cognition, behavior, emotion, and the pathophysiology of Alzheimer’s disease and autism spectrum disorder^13–18^. In postmortem studies in humans, decreased aromatase immunoreactivity was observed in the hypothalamus of the patients with major depressive disorder^19^ and Alzheimer’s disease^20^.

We had previously developed [^11^C]cetrozole as a PET tracer for aromatase^21^. [^11^C]Cetrozole shows better specificity and selectivity for aromatase than the previously developed [^11^C]vorozole^22^. Furthermore, the radioactive metabolites of [^11^C]cetrozole were not taken up into the brain, unlike the metabolite of [^11^C]vorozole, indicating that [^11^C]cetrozole could be used for highly quantitative measurement of aromatase in the brain. Human PET studies with [^11^C]cetrozole were performed in healthy participants^23,24^ and demonstrated the association between aromatase levels in the brain and human personality^24^.

**Figure 1:**
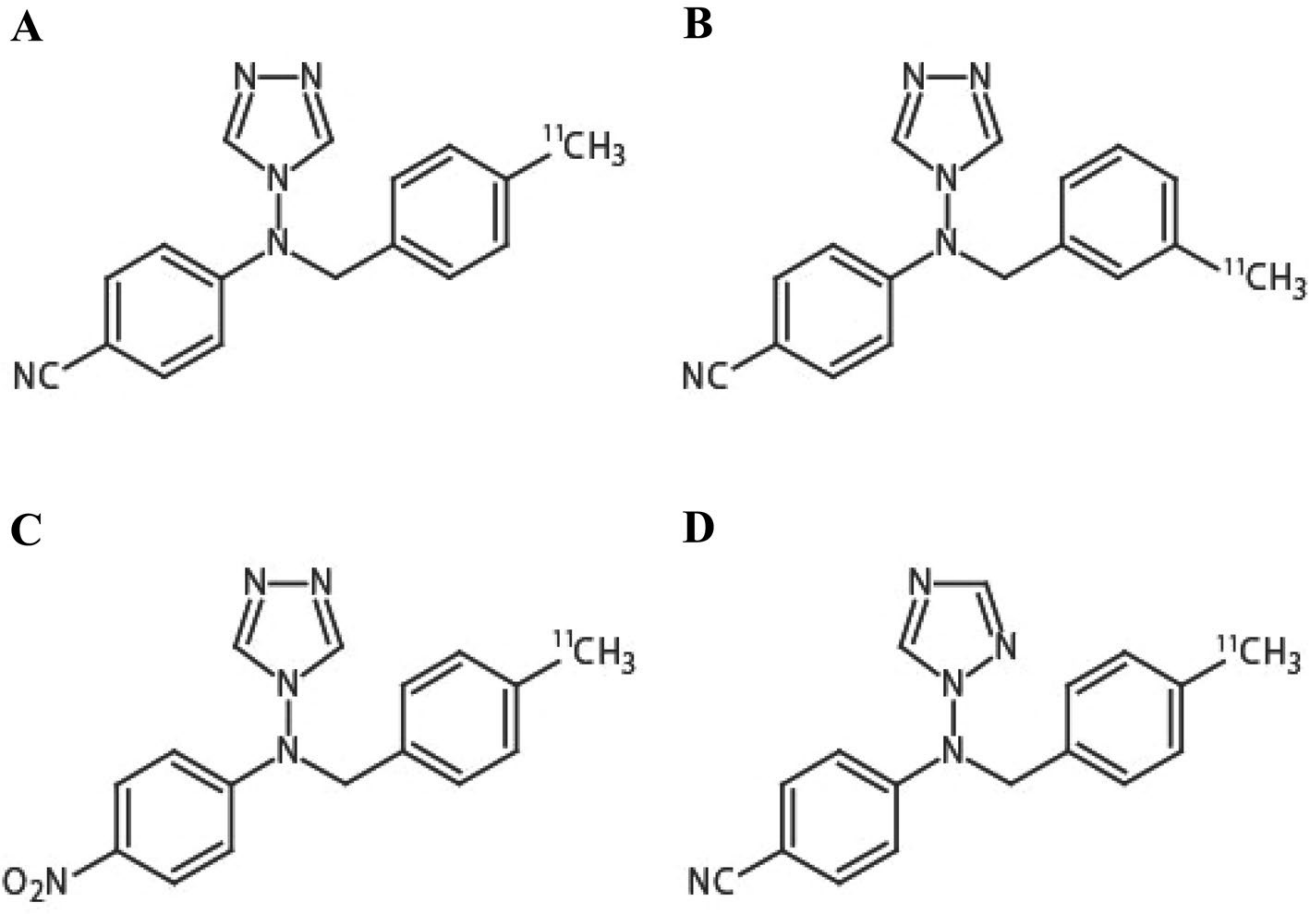
The chemical structures of [^11^C]cetrozole (**A**) and its analogs, [^11^C]meta-cetrozole (**B**), [^11^C]nitro-cetrozole (**C**), and [^11^C]iso-cetrozole (**D**). The methyl moiety in [^11^C]meta-cetrozole showed a different position from that in [^11^C]cetrozole. [^11^C]Nitro-cetrozole contained a nitro group instead of the cyano group of [^11^C]cetrozole. [^11^C]Iso-cetrozole showed a different nitrogen position in the triazole in comparison with [^11^C]cetrozole.

To develop more efficient PET tracer(s) for aromatase imaging in the human brain, we synthesized three analogs of cetrozole: meta-cetrozole, nitro-cetrozole^25^ and iso-cetrozole^26^ previously referred to as TMD-322^27^ (Fig. 1). These analogs differed from cetrozole in terms of the position of the methyl group, replacement of the cyano group with a nitro group, or the positioning of one nitrogen atom in triazole, respectively. The inhibitory activities of these three analogs toward aromatase were evaluated, and PET imaging of brain aromatase was performed using the corresponding ^11^C-labeled tracers in nonhuman primates. Iso-cetrozole, which was the most promising analog in a monkey PET study, was evaluated in the present human PET study and compared with the previous human PET study with [^11^C]cetrozole.

## Results

### Aromatase inhibitory activity

Aromatase inhibitory activity was measured using marmoset placenta homogenate with unlabeled meta-cetrozole, nitro-cetrozole, iso-cetrozole, and cetrozole. IC_50_ values were 3.50, 0.73, 0.68, and 0.98 nM for meta-cetrozole, nitro-cetrozole, iso-cetrozole, and cetrozole, respectively (Supplemental Fig. S22).

### Animal PET studies

**Figure 2:**
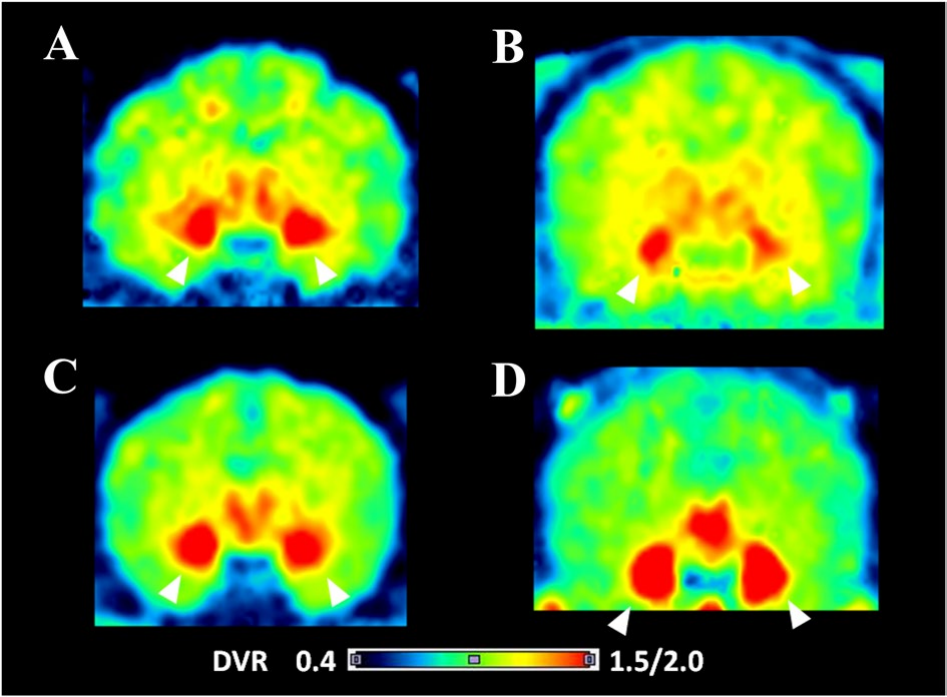
The distribution volume ratio images of [^11^C]cetrozole (**A**), [^11^C]meta-cetrozole (**B**), [^11^C]nitro-cetrozole (**C**), and [^11^C]iso-cetrozole (**D**) in rhesus monkey brain (coronal section). The slices contain the amygdala indicated by arrowhead. The scale ranges of the color bar are 0.4–1.5 for [^11^C]cetrozole, [^11^C]meta-cetrozole, and [^11^C]nitro-cetrozole, and 0.4–2.0 for [^11^C]iso-cetrozole.

**Figure 3:**
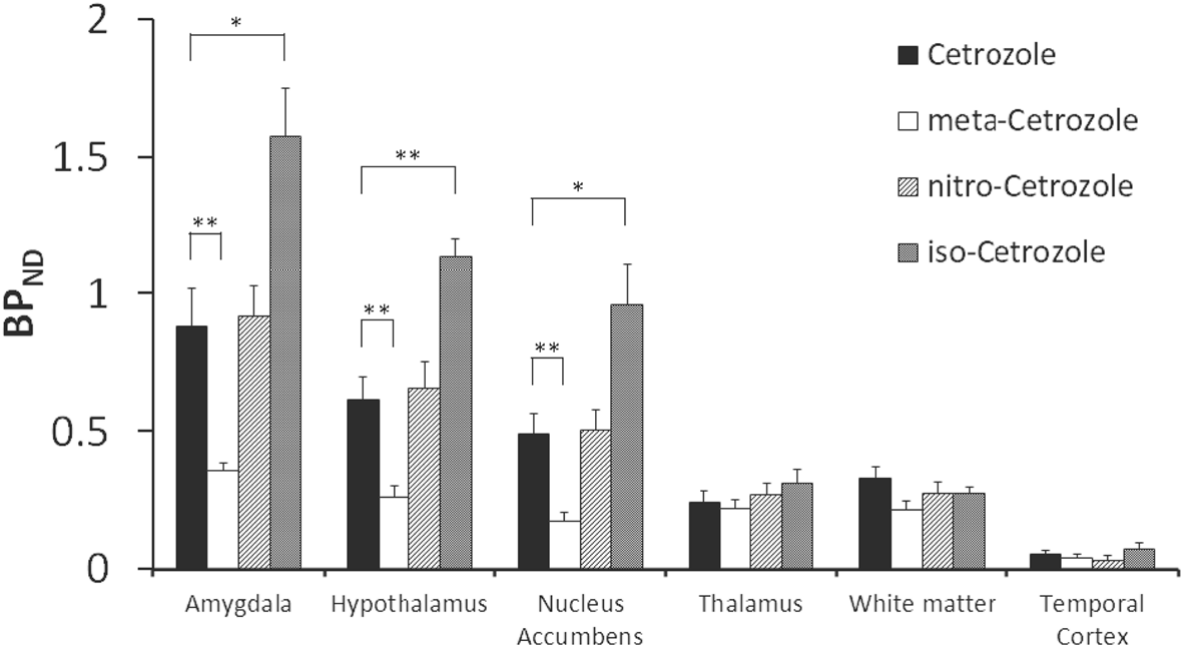
BP_ND_ values in the amygdala, hypothalamus, nucleus accumbens, and the white matter of [^11^C]cetrozole, [^11^C]meta-cetrozole, [^11^C]nitro-cetrozole, and [^11^C]iso-cetrozole (N = 4, mean ± S.E.) in rhesus monkey brain. [^11^C]Meta-cetrozole showed a lower BP_ND_ than [^11^C]cetrozole in the amygdala, hypothalamus, and nucleus accumbens (**P < 0.01). [^11^C]Iso-cetrozole showed a higher BP_ND_ than [^11^C]cetrozole in the amygdala, nucleus accumbens (*P < 0.05), and hypothalamus (**P < 0.01).

The distribution volume ratio (DVR) images of all tracers showed a similar distribution pattern, i.e., high binding of the tracers was observed in the amygdala, hypothalamus, and nucleus accumbens; however, the signal intensity was different (Fig. 2). The images of [^11^C]iso-cetrozole showed the highest-intensity signals among the tracers. Nondisplaceable binding potential (BP_ND_) in the amygdala, hypothalamus, nucleus accumbens, thalamus, white matter, and temporal cortex were calculated using the superior semilunar lobule of cerebellum as a reference region with the four tracers, as shown in Fig. 3. The BP_ND_ values of [^11^C]cetrozole and [^11^C]nitro-cetrozole were comparable. BP_ND_ of [^11^C]meta-cetrozole was significantly lower than that of [^11^C]cetrozole in the aromatase-rich regions (amygdala, *P* < 0.01; hypothalamus, *P* < 0.01; nucleus accumbens, *P* < 0.01). BP_ND_ of [^11^C]iso-cetrozole was 178–195% higher than that of [^11^C]cetrozole in the aromatase-rich regions (amygdala, *P* < 0.05; hypothalamus, *P* < 0.01; nucleus accumbens, *P* < 0.05). All tracers showed low binding to the nonspecific binding region of the thalamus, white matter, and temporal cortex in rhesus monkey brain.

**Figure 4:**
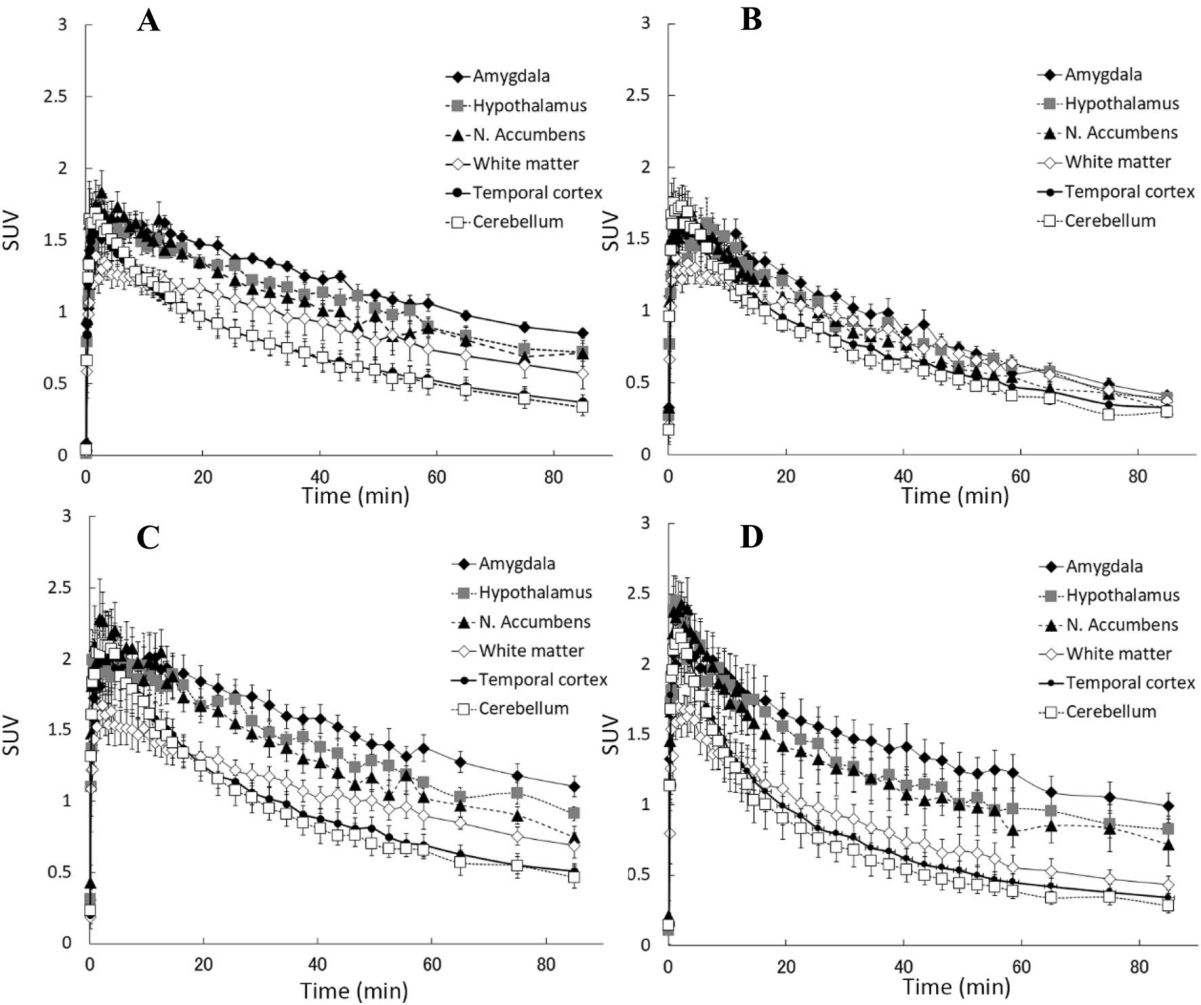
Time-activity curves of [^11^C]-cetrozole (**A**), [^11^C]meta-cetrozole (**B**), [^11^C]nitro-cetrozole (**C**), and [^11^C]iso-cetrozole (**D**) in rhesus monkey brain (N = 4, mean ± S.E.). Aromatase-rich regions (amygdala, hypothalamus, and nucleus accumbens), nonspecific region (white matter), and reference region for Logan reference tissue model analysis (cerebellum) are shown.

The time–activity curves of all tracers showed a time-dependent gradual decline in the accumulated regions (Fig. 4). The curves for [^11^C]cetrozole, [^11^C]nitro-cetrozole, and [^11^C]iso-cetrozole showed higher accumulation of tracers in the aromatase-rich regions (amygdala, hypothalamus, and nucleus accumbens) than in the aromatase-less region (cerebellum). In contrast, the gap in the curves between the aromatase-rich and aromatase-less regions was small for [^11^C]meta-cetrozole.

### Human studies

**Figure 5:**
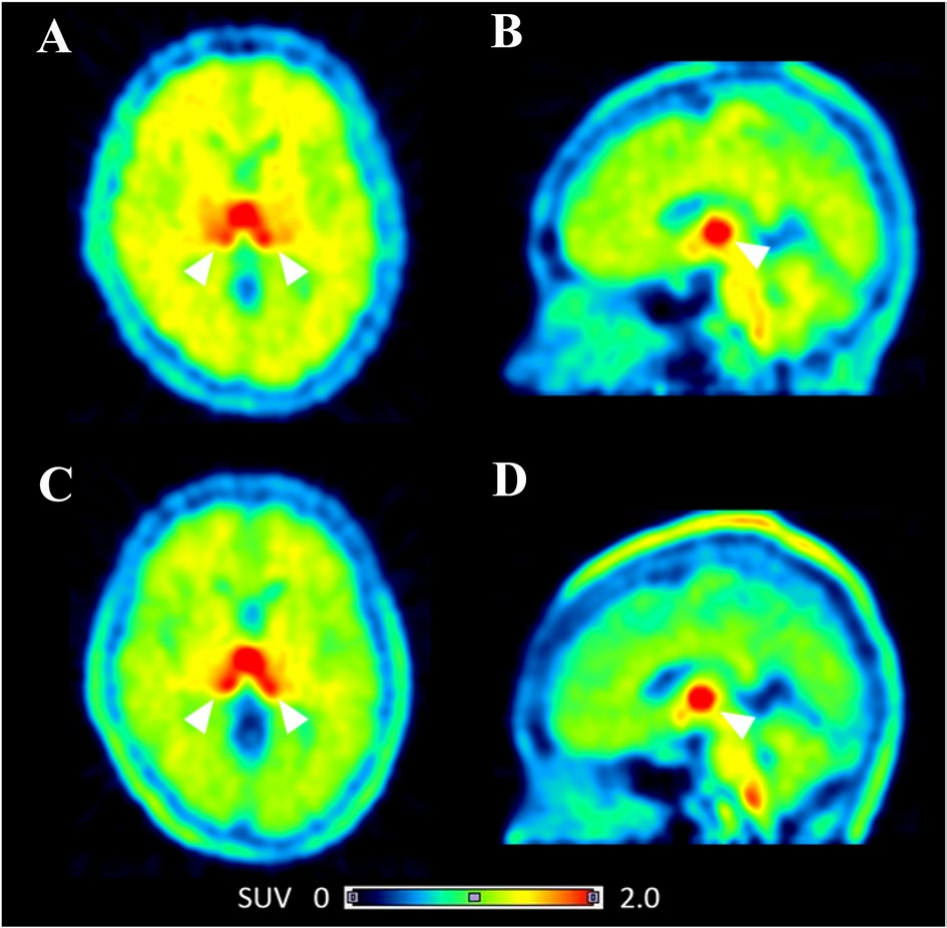
The representative SUV images of [^11^C]cetrozole (**A**,**B**) and [^11^C]iso-cetrozole (**C**,**D**) in the brains of similar individuals (**A**,**C** transaxial slices; **B**,**D** sagittal slices). Arrow heads indicate the thalamus.

**Figure 6:**
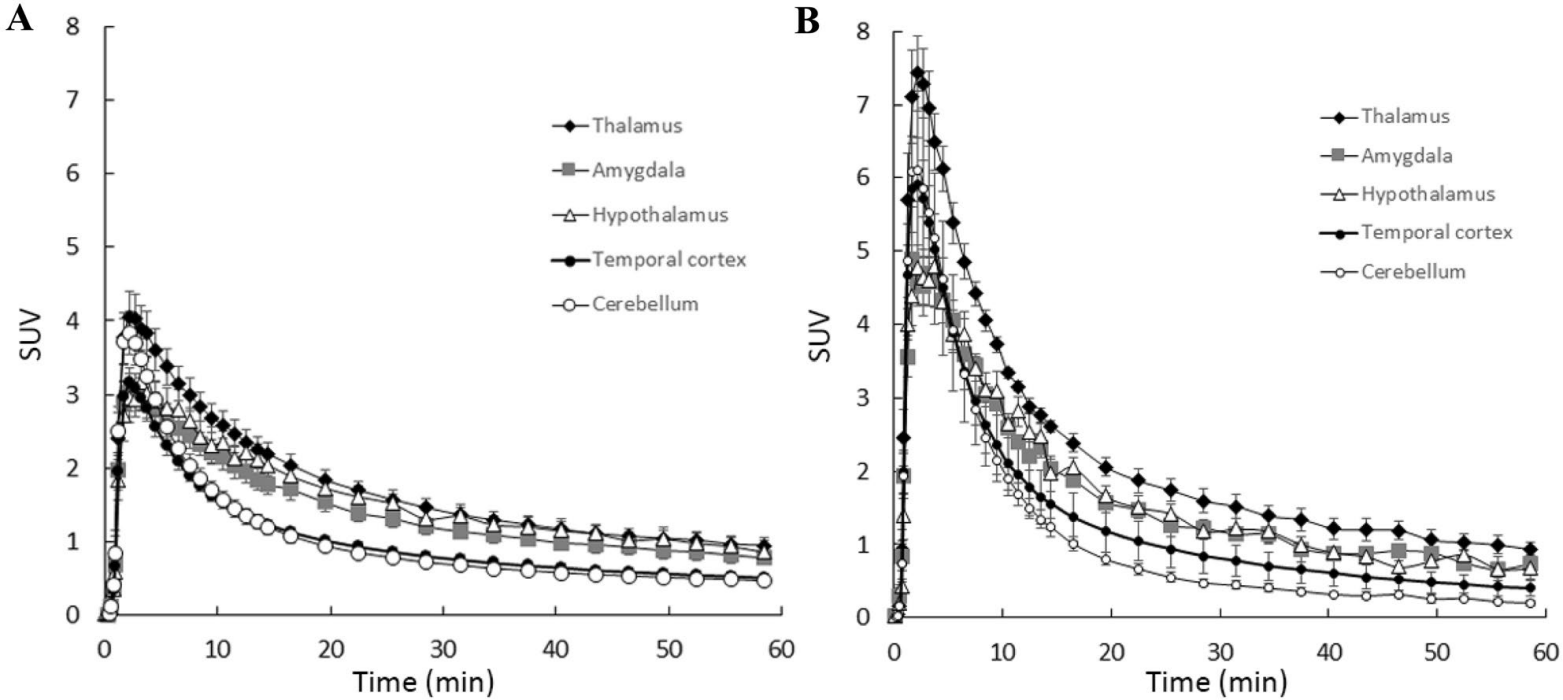
Time-activity curves of [^11^C]cetrozole (**A**, N = 21) and [^11^C]iso-cetrozole (**B**, N = 6) in the human brain (mean ± S.E.). The aromatase-rich regions (thalamus, amygdala, and hypothalamus) and the reference region for Logan reference tissue model analysis (cerebellum) are shown. The [^11^C]iso-cetrozole curves demonstrate rapid clearance from the tissues in comparison with cetrozole.

**Figure 7:**
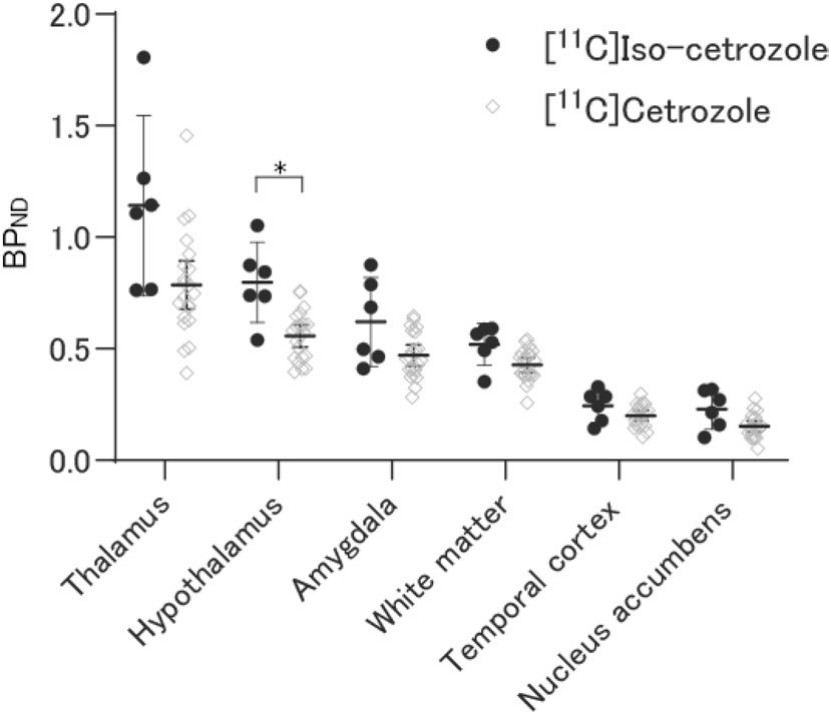
BP_ND_ values in the thalamus, hypothalamus, amygdala, white matter, temporal cortex, and nucleus accumbens of [^11^C]cetrozole (N = 21) and [^11^C]iso-cetrozole (N = 6) in the human brain. Each dot indicates individual value. Mean and 95% confidence intervals are also shown. In the hypothalamus, [^11^C]iso-cetrozole showed significantly higher BP_ND_ than [^11^C]cetrozole. No significant difference was observed between the BP_ND_ of [^11^C]iso-cetrozole and [^11^C]cetrozole in the other regions.

**Figure 8:**
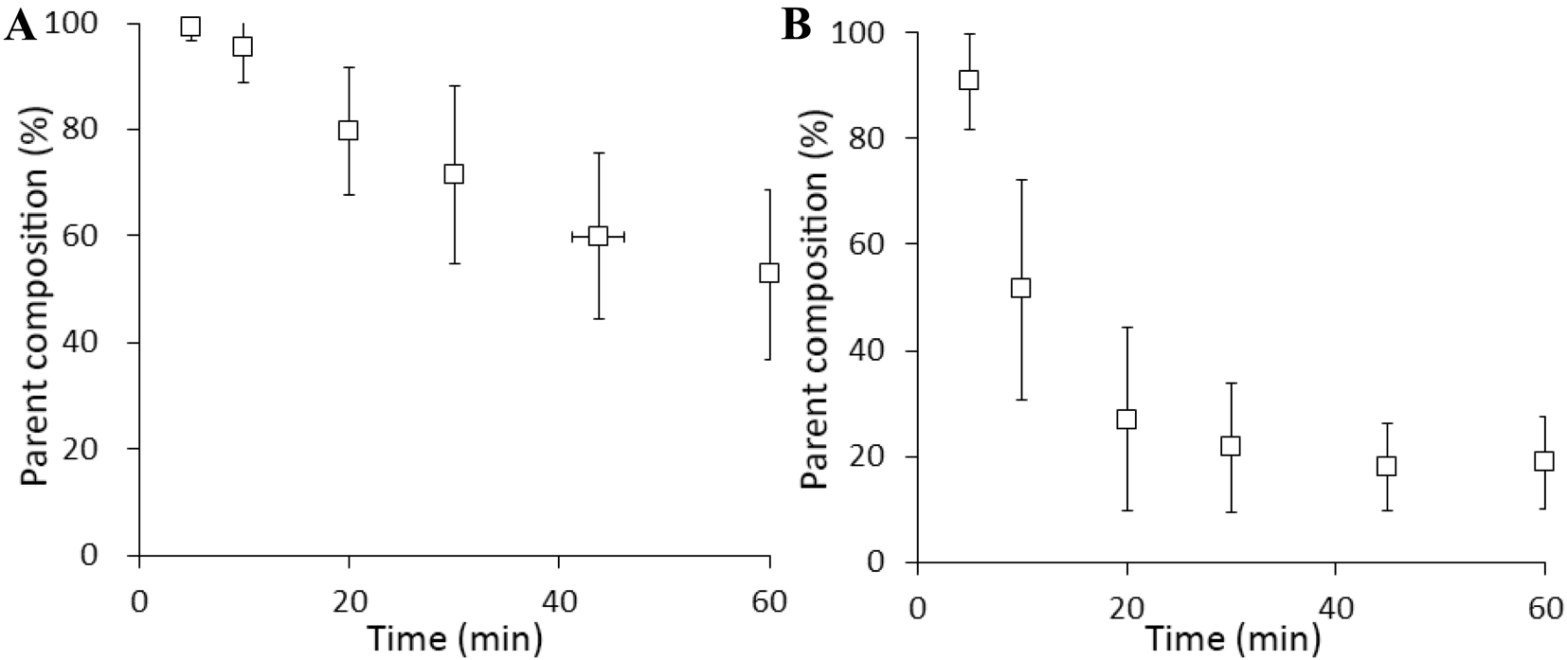
Parent composition of [^11^C]cetrozole (**A**, N = 21) and [^11^C]iso-cetrozole (**B**, N = 5) in human plasma (mean ± SD). The parent fraction of [^11^C]cetrozole and [^11^C]iso-cetrozole remained 80% and 27%, respectively, at 20 min after the injection.

Human PET studies were performed with [^11^C]iso-cetrozole and the data were compared with the previously published results for [^11^C]cetrozole^24^. The distribution pattern of [^11^C]iso-cetrozole was similar to that of [^11^C]cetrozole in humans (Fig. 5). High binding of [^11^C]iso-cetrozole was observed in the amygdala, hypothalamus, thalamus, and medulla. The time–activity curves of both tracers are shown in Fig. 6. The time–activity curves of [^11^C]iso-cetrozole demonstrate relatively quick clearance from tissues in comparison with [^11^C]cetrozole. Both tracers showed faster clearance from tissues in humans in comparison with their clearance in rhesus monkeys (Fig. 4). In calculations performed with the Logan reference tissue model, [^11^C]iso-cetrozole showed higher BP_ND_ values in the hypothalamus than [^11^C]cetrozole (*P* < 0.05); however, the two tracers did not show differences in the BP_ND_ values in the thalamus, amygdala, white matter, temporal cortex, and nucleus accumbens (Fig. 7). The measurement of [^11^C]iso-cetrozole metabolites in the plasma revealed the relatively quick metabolism of this tracer (Fig. 8). The proportions of the parent compound of [^11^C]iso-cetrozole remained at 27% and 19% at 20 and 60 min, respectively, after injection.

## Discussion

In this study, we prepared three analogs of [^11^C]cetrozole to determine the significant structural factors for a potent PET tracer for brain aromatase imaging (Fig. 1). We also aimed to develop a more potent tracer than [^11^C]cetrozole^21,24^ that could enable more precise analysis of aromatase expression in the human brain. The three analogs were [^11^C]meta-cetrozole, [^11^C]nitro-cetrozole, and [^11^C]iso-cetrozole. The inhibitory activity of the analogs including cetrozole toward aromatase in the microsomal fraction of marmoset placenta was in the following order: iso-cetrozole (IC_50_ = 0.68 nM), nitro-cetrozole (IC_50_ = 0.73), cetrozole (IC_50_ = 0.98), and meta-cetrozole (IC_50_ = 3.50). This result indicated that (1) the methyl group substituted at the *para* position of the benzene ring is important, (2) the cyano group can be replaced with other electron-withdrawing groups without a significant decrease in the inhibitory activity, and (3) the triazole moiety can be exchanged with other types of triazoles. These features were consistent with the results reported for the structure–activity relationship of YM511^25,27^, which is a leading aromatase inhibitor of cetrozole. To examine the potential of each analog as a PET tracer, ^11^C-labeled analogs were prepared by palladium(0)-mediated rapid ^11^C-methylation^28^ from the corresponding tributylstannyl precursors.

PET studies with rhesus monkeys were conducted using three tracers and the data were compared with the previous results using [^11^C]cetrozole. All analog tracers penetrated the blood–brain barrier and showed a distribution pattern similar to that of [^11^C]cetrozole. However, the binding properties of the analogs were somewhat different from those of [^11^C]cetrozole. [^11^C]Meta-cetrozole showed low BP_ND_, which was 35–43% of that of [^11^C]cetrozole in the aromatase-rich regions (Fig. 2B vs. A). [^11^C]Nitro-cetrozole had comparable binding ability to [^11^C]cetrozole (Fig. 2C vs. A). [^11^C]Iso-cetrozole had higher BP_ND_ than the other analogs and [^11^C]cetrozole (Fig. 2D vs. A). This variation in BP_ND_ between analogs was in accordance with the order of magnitude of IC_50_ values of analogs.

Since the rhesus monkey PET study demonstrated the high potential of [^11^C]iso-cetrozole for imaging and quantitation of brain aromatase, we performed a human PET study. Six healthy volunteers (three females and three males) were recruited for 60-min PET scans with [^11^C]iso-cetrozole. The distribution pattern of [^11^C]iso-cetrozole was similar to that of [^11^C]cetrozole, suggesting that [^11^C]iso-cetrozole binds to aromatase in the human brain (Fig. 5). However, unlike the rhesus monkey study, [^11^C]iso-cetrozole showed higher BP_ND_ than [^11^C]cetrozole only in the hypothalamus (Fig. 7). The time–activity curves of [^11^C]iso-cetrozole showed a relatively rapid decline, indicating that [^11^C]iso-cetrozole showed higher susceptibility to metabolism than [^11^C]cetrozole (Fig. 8). The parent fraction of [^11^C]iso-cetrozole remained at 27% and 19% at 20 and 60 min, respectively, after administration. In contrast, the parent fraction of [^11^C]cetrozole remained at 80% and 53% at 20 and 60 min, respectively, after administration. These observations are consistent with the results of a cassette-microdose clinical study in which we administered cetrozole and iso-cetrozole intravenously or orally to healthy participants^27^. The cassette-microdose study showed that total body clearance and bioavailability were 12.1 mL/min/kg and 34.9% for cetrozole, and 16.8 mL/min/kg and 18.4%, respectively, for iso-cetrozole. The underlying mechanisms remain unknown, however, it might be caused by the hepatic CYP-mediated metabolism. CYP2C19 had high metabolic activities against cetrozole, in the meanwhile, not only CYP2C19 but CYP1A2 and CYP3A4 showed rapid velocity of metabolism against iso-cetrozole^27^. A desirable molecular imaging probe should have distinctive characteristics such as high stability in vivo to ensure the quantitative measurments^29^. [^11^C]cetrozole has preferable metabolic stability for brain aromatase imaging in humans, although [^11^C]iso-cetrozole might also be useful to measure aromatase level in living human brain because of its high binding potential.

In the present study, we developed three analogs of cetrozole, namely, meta-cetrozole, nitro-cetrozole, and iso-cetrozole, to identify a more efficient PET tracer for aromatase imaging in the human brain. [^11^C]Iso-cetrozole showed high binding potential in the rhesus monkey brain; however, it did not function similarly in the human. From this perspective, PET is a significant tool that allows us to investigate molecular dynamics in living humans. By using [^11^C]cetrozole/[^11^C]iso-cetrozole and PET techniques, the mechanism of brain functions and diseases in which aromatase is involved in humans might be clarified in the near future.

## Materials and methods

### Synthesis of cetrozole analogs and their tributylstannyl precursors for ^11^C-labeled PET tracers

Detailed synthetic procedures are provided in the Supplemental Data.

### Synthesis of ^11^C-labeled cetrozole analogs

^11^C-radiolabeling of meta-cetrozole, nitro-cetrozole, and iso-cetrozole was achieved by methods similar to those used our previous report on [^11^C]cetrozole^21,24^, which involved palladium-mediated ^11^C-methylation using the corresponding tri-*n*-butylstannane precursors^28,30^. After radiopharmaceutical formulation for the in vivo PET study, the molar activities were 30–134, 44–81, and 42–170 GBq/μmol for ^11^C-labeled meta-cetrozole, nitro-cetrozole, and iso-cetrozole, respectively. The radiochemical purity of all radiotracers was greater than 99%. The chemical purity of all radiotracers was regularly greater than 95%; however, some chemical experiments yielded 80% purity once in the synthesis of [^11^C]meta-cetrozole and 84–88% purities twice in the synthesis of [^11^C]iso-cetrozole. Detailed radiochemistry is described in the Supplemental Data.

### Aromatase inhibitory activity

The aromatase inhibitory activity assay using marmoset placenta was performed by the same protocols as previously described^21^. Briefly, the incubation mixture contained 110–120 nM [4-^14^C]testosterone (1.96 GBq/mmol, GE Healthcare Japan, Tokyo, Japan), 0.24 mM NADPH (Sigma-Aldrich, St. Louis, MO, US), 0.3, 1, 3, 10, or 30 nM unlabeled cetrozole, meta-cetrozole, nitro-cetrozole, or iso-cetrozole, and 10 μL of the microsomal fraction of marmoset placenta in a total volume of 400 μL at 37 °C. The mixture was centrifuged and the aqueous phase was evaporated. The residue was dissolved in ethyl acetate, and aliquots were applied to Silica 60 thin-layer chromatography plates (Merck Millipore, Burlington, MA, US). The plates were developed with ethyl acetate/isooctane. After migration, the plates were dried and exposed to BAS-SR2040 imaging plates overnight. The distribution of radioactivity on the imaging plates was determined with digital PSL autoradiography.

### Animals

Male adult rhesus monkeys (*Macaca mulatta*; 3.9–7.7 kg) were housed individually and received food twice a day and water ad libitum. Animals were maintained and handled in accordance with the recommendations of the United States National Institutes of Health, and the study was approved by the Animal Care and Use Committee of Kobe Institute at RIKEN.

### Animal PET studies

PET scans were performed in rhesus monkeys by using [^11^C]meta-cetrozole, [^11^C]nitro-cetrozole, and [^11^C]iso-cetrozole (N = 4 each). The monkeys were sedated with ketamine hydrochloride (15 mg/kg, i.m.), and venous cannulae were placed in the saphenous veins for further continuous anesthesia with propofol (10 mg/kg/h) and PET tracer injection. Before the emission scan, a transmission scan was performed for 30 min for attenuation correction. Each tracer ([^11^C]meta-cetrozole, 153–310 MBq; [^11^C]nitro-cetrozole, 219–292 MBq; [^11^C]iso-cetrozole, 214–345 MBq) was administered intravenously as a bolus. The monkeys were scanned for 90 min in list mode with the PET scanner of Focus220 (Siemens, Knoxville, TN, US). The acquired data were sorted into dynamic sinograms (4 × 30 s, 3 × 60 s, 2 × 150 s, 2 × 300 s, and 7 × 600 s) and reconstructed using Fourier Rebinninig (FORE) and 2D-filtered back projection (FBP) with a Hann filter and a cutoff frequency of 0.4 cycle per pixel.

### Analysis of animal PET data

For PET image analysis, we employed same modeling and protocol as [^11^C]cetrozole analysis reported previously^21^. Briefly, using PMOD software (PMOD Technologies Ltd., Zurich, Switzerland), volumes of interest (VOIs) were delineated in the superior semilunar lobule of cerebellum, amygdala, hypothalamus, nucleus accumbens, white matter, thalamus and temporal cortex. The data were analyzed with Logan’s reference tissue model based on the averaged k2′^31^ values. The averaged k2′ values were calculated in the aromatase-rich regions, namely amygdala and hypothalamus, with simplified reference tissue model^32^ using the superior semilunar lobule of cerebellum as a reference region. Then, BP_ND_ and DVR were calculated. The difference in BP_ND_ between the tracers was analyzed statistically using the Mann–Whitney *U* test.

### Human participants

We recruited six healthy volunteers (three females and three males, average age of 38.0 ± 1.0, and 38.0 ± 6.9 y.o., mean ± SD, respectively) for the PET study with [^11^C]iso-cetrozole. All participants underwent a brain PET scan, and two of the three females (38 and 39 years old) underwent a whole-body PET scan to measure their radiation exposure. Two females and two males had undergone a brain PET scan with [^11^C]cetrozole in a previous study (3.3–3.9 yr previously). All participants provided written informed consent.

### Human PET studies

The human PET studies were performed by the same protocols as human PET studies with [^11^C]cetrozole which were previously described^24^. The participants lay down in the PET scanner (Biograph-16, Siemens, Knoxville, TN, US) with their heads fixed with bandages to minimize movement. The left and right median cubital veins were cannulated for blood sampling and radiotracer administration, respectively. CT scans were carried out for head positioning and attenuation correction before the emission scans. At the start of the emission scan, [^11^C]iso-cetrozole (201–309 MBq) was intravenously administered for approximately 30 s, and the catheter line was flushed with 15–20 mL saline to prevent radiotracer retention. Serial PET scanning of the brain was performed for 60 min in the list mode and sorted into dynamic sinograms (6 × 10 s, 6 × 30 s, 11 × 60 s, and 15 × 180 s). Images were reconstructed with FORE and FBP with no post filter. Blood samples were taken from the venous line at 5, 10, 20, 30, 45, and 60 min after administration of [^11^C]iso-cetrozole, and used for radiometabolite analyses (N = 5). One sample was missed because blood could not be collected from one person.

### Analysis of human PET data

For quantitative analyses, PMOD software was used. VOIs were delineated in the thalamus, amygdala, and hypothalamus, which are known to contain a rich supply of aromatase enzyme^33–35^, and in the superior semilunar lobule of cerebellum, temporal cortex and nucleus accumbens. Decay-corrected time-activity curves were generated for each brain region. The data were analyzed with a Logan reference tissue model based on the k2′ value. The k2′ values were calculated in the aromatase-rich region, namely thalamus, with simplified reference tissue model^32^ using the superior semilunar lobule of cerebellum as a reference, and BP_ND_ and DVR were calculated. A 95% confidence interval was calculated to evaluate the difference in BP_ND_ between the tracers.

### Radiometabolite analysis in plasma (rhesus monkey and human)

The radiometabolite analysis in plasma was performed by the same protocols as previously described^21,24^. Briefly, the collected blood samples were deproteinated and centrifuged. The supernatants were subjected to thin-layer chromatography using RP-18 plates (Merck Millipore). The plates were developed with acetonitrile/water/formic acid (50:50:0.75). After migration, the plates were exposed to BAS TR2040 imaging plates (Fuji Photo Film Co., Tokyo, Japan) for 40 min. The distribution of radioactivity on the imaging plates was determined with digital PSL autoradiography using a Fuji FLA-7000 analyzer, and the data were analyzed using the MultiGauge image analysis program (Fuji Photo Film Co.).

### Data for [^11^C]cetrozole

In this study, the data of [^11^C]cetrozole in monkeys were originally published in *JNM*. Takahashi et al. ^11^C-Cetrozole: An improved C-^11^C-methylated PET probe for aromatase imaging in the brain. *J Nucl Med*. 2014;55:852–857^21^. The data of [^11^C]cetrozole in humans were published in *Scientific Reports*. Takahashi K et al. Association between aromatase in human brains and personality traits. *Sci Rep*. 2018;8:16841^24^.

### Ethics approval

The protocol was approved by the Ethics Committee of Kobe Institute of RIKEN and Osaka City University Graduate School of Medicine. All experiments were conducted in compliance with national legislation and the Code of Ethical Principles for Medical Research Involving Human Subjects of the World Medical Association (*Declaration of Helsinki*) and registered in the UMIN Clinical Trials Registry (No. UMIN000006586). The study was carried out in compliance with the ARRIVE guidelines.

## Supplementary Information

Supplementary Information.
