# Supplementary material for: PET imaging of brain aromatase in humans and rhesus monkeys by 11C-labeled cetrozole analogs
Source: Sci Rep. 2021 Dec 8;11:23623. doi: 10.1038/s41598-021-03063-8 (PMC8654920; doi:10.1038/s41598-021-03063-8)
Supplement: Supplementary file 1 — Supplementary Information. [file 41598_2021_3063_MOESM1_ESM.docx]

**Supplemental data**

***Chemical synthesis***

*General remarks*

4-(4*H*-1,2,4-Triazol-4-ylamino)benzonitrile (**1**) was prepared according to the reported method^1^ by the reaction of 4-fluorobenzonitrile (**13**) and 4-amino-4*H*-1,2,4-triazole (**9**) in DMSO in the presence of potassium *tert*-butoxide. 4-(Tributylstannyl)benzyl bromide was prepared according to the reported method.^2^

4-Fluorobenzonitrile (**13**), 4-amino-4*H*-1,2,4-triazole (**9**), potassium *tert*-butoxide, potassium carbonate, 3-bromobenzyl alcohol (**4**), tributyltin(IV) chloride, triethylamine, methanesulfonyl chloride, sodium bromide, 4-fluoronitrobenzene (**8**), 4-methylbenzyl bromide, potassium hydroxide, and hydroxylamine-*O*-sulfonic acid were purchased from Wako Pure Chemical Industries Ltd., Japan. 3-Methylbenzyl bromide and 1,2,4-triazole (**18**) were purchased from Sigma-Aldrich Japan K.K. A solution of *n*-butyllithium in *n*-hexane was purchased from Kanto Chemical Co., Inc., Japan. All other chemical reagents used were commercial grade and used as received.

Analytical thin-layer chromatography (TLC) was performed on precoated (0.25 mm) silica-gel plates (Merck Chemicals, Silica Gel 60 F_254_). Flash column chromatography was conducted using silica-gel (Kanto Chemical Co., Inc., Silica Gel 60N, spherical neutral, particle size 40–50 μm or particle size 63–210 μm). Melting points (Mp) were measured on a YANACO MP-J3 instrument and are uncorrected. ^1^H and ^13^C NMR spectra were obtained with a Bruker AVANCE 500 spectrometer at 500 and 125 MHz, respectively. CDCl_3_ (KANTO) and DMSO-*d*_6_ (KANTO) were used as solvents for obtaining NMR spectra. Chemical shifts (δ) are given in parts per million (ppm) downfield from (CH_3_)_4_Si (δ 0.00 for ^1^H NMR in CDCl_3_) or the solvent peak (δ 2.49 for ^1^H NMR and δ 39.5 for ^13^C NMR in DMSO-*d*_6_, and δ 77.0 for ^13^C NMR in CDCl_3_) as an internal reference with coupling constants (*J*) in hertz (Hz). The abbreviations s, d, t, and m signify singlet, doublet, triplet, and multiplet, respectively. IR spectra were measured by diffuse reflectance method on a SHIMADZU IRPrestige-21 spectrometer attached with DRS-8000A with the absorption band given in cm^–1^. Elemental analyses were performed with a YANACO CHN CORDER MT-5 at the Center for Advanced Materials Analysis (Suzukakedai), Technical Department, Tokyo Institute of Technology. High-resolution mass spectra (HRMS) were measured on a JEOL JMS-700 mass spectrometer under positive fast atom bombardment (FAB^+^) conditions at the Center for Advanced Materials Analysis (Suzukakedai), Technical Department, Tokyo Institute of Technology. The authors thank Ms. Masayo Ishikawa for HRMS analysis.

*Synthesis of 4-((3-methylbenzyl)(4H-1,2,4-triazol-4-yl)amino)benzonitrile (****2****, meta-cetrozole) and 4-((4H-1,2,4-triazol-4-yl)(3-(tributylstannyl)benzyl)amino)benzonitrile (****3****, precursor of [^11^C]meta-cetrozole)*

**Supplemental Fig S1.** Synthetic scheme of meta-cetrozole (**2**) and precursor of [^11^C]meta-cetrozole.

4-((3-Methylbenzyl)(4*H*-1,2,4-triazol-4-yl)amino)benzonitrile (**2**, meta-cetrozole)

Under argon atmosphere, a mixture of 4-(4*H*-1,2,4-triazol-4-ylamino)benzonitrile (**1**) (300 mg, 1.62 mmol), 3-methylbenzyl bromide (265 μL, 1.96 mmol), and potassium carbonate (448 mg, 3.24 mmol) in acetone (15 mL) was stirred at room temperature for 5 h. To this was added water (50 mL) and the mixture was extracted with CH_2_Cl_2_ (50 mL × 3). The combined organic extracts were successively washed with water (50 mL × 1) and brine (50 mL × 1), dried (Na_2_SO_4_), filtered, and concentrated under reduced pressure. The residue was purified by flash column chromatography (silica-gel 30 g, CH_2_Cl_2_/CH_3_OH = 9/1) to give 4-((3-methylbenzyl)(4*H*-1,2,4-triazol-4-yl)amino)- benzonitrile (**2**, meta-cetrozole) (373 mg, 79.6%) as a colorless solid. Recrystallization from EtOAc (50 mL) and *n*-hexane (25 mL) afforded colorless plates (293 mg, 62.5%); TLC *R*_f_ = 0.41 (CH_2_Cl_2_/CH_3_OH = 9/1); Mp 185–186 ˚C; ^1^H NMR (500 MHz, DMSO-*d*_6_) δ 2.26 (s, 3H, CH_3_), 5.01 (s, 2H, benzylic CH_2_), 6.72–6.74 (AA’BB’, 2H, aromatic), 7.07 (d, 1H, *J* = 7.8 Hz, aromatic), 7.09 (d, 1H, *J* = 7.8 Hz, aromatic), 7.12 (s, 1H, aromatic), 7.19 (dd, 1H, *J* = 7.8, 7.8 Hz, aromatic), 7.74–7.75 (AA’BB’, 2H, aromatic), 8.79 (s, 2H, triazole); ^13^C NMR (125 MHz, DMSO-*d*_6_) δ 20.9, 57.2, 102.7, 113.6 (2C), 119.0, 125.4, 128.5, 128.7, 128.9, 133.9 (2C), 134.7, 137.9, 143.4 (2C), 151.6; IR (KBr, cm^–1^) 513, 546, 615, 648, 669, 704, 737, 766, 791, 827, 860, 949, 1007, 1040, 1069, 1140, 1182, 1213, 1300, 1321, 1391, 1425, 1460, 1510, 1605, 1692, 2218, 2868, 2918, 3011, 3051, 3100, 3121; Anal. Calcd. for C_17_H_15_N_5_: C, 70.57; H, 5.23; N, 24.21. Found: C, 70.33; H, 5.30; N, 23.94.

3-(Tributylstannyl)benzyl alcohol (**5**)^3^

Under argon atmosphere, to a solution of 3-bromobenzyl alcohol (**4**) (2.66 mL, 22.2 mmol) in anhydrous THF (150 mL) was slowly added a solution of *n*-butyllithium (1.63 M, 30.0 mL, 48.9 mmol) in *n*-hexane at –78 °C. After stirring the mixture for 30 min at the same temperature, to this was added tributyltin(IV) chloride (13.5 mL, 49.8 mmol) at –78 °C and the mixture was allowed to warm to room temperature. After stirring for 1 h, the reaction mixture was concentrated under reduced pressure. To the residue was added water (200 mL) and the mixture was extracted with CH_2_Cl_2_ (80 mL × 3). The combined organic extracts were successively washed with water (100 mL × 3) and brine (100 mL × 1), dried (Na_2_SO_4_), filtered, and concentrated under reduced pressure. The residue was purified by flash column chromatography (silica-gel 220 g, *n*-hexane/EtOAc = 10/1) to give 3-(tributylstannyl)benzyl alcohol (**5**) (7.47 g, 84.8%) as a colorless oil; TLC *R*_f_ = 0.52 (*n*-hexane/EtOAc = 4/1), *R*_f_ = 0.52 (*n*-hexane/CH_2_Cl_2_/EtOAc = 5/2/1); ^1^H NMR (500 MHz, CDCl_3_) δ 0.89 (t, 9H, *J* = 7.3 Hz, 3CH_3_), 0.99–1.12 (m, 6H, 3CH_2_), 1.29–1.37 (m, 6H, 3CH_2_), 1.51–1.58 (m, 6H, 3CH_2_), 4.68 (d, 2H, *J* = 6.0 Hz, benzylic CH_2_), 7.29–7.48 (m, 4H, aromatic) (The signal for the proton of the hydroxy group was not observed); ^13^C NMR (125 MHz, CDCl_3_) δ 9.5 (3C, ^1^*J*(^119^Sn-^13^C) = 338.0 Hz, ^1^*J*(^117^Sn-^13^C) = 323.2 Hz), 13.7 (3C), 27.4 (3C, ^3^*J*(^119/117^Sn-^13^C) = 56.2 Hz), 29.1 (3C, ^2^*J*(^119/117^Sn-^13^C) = 19.9 Hz), 65.7, 126.8, 128.0 (^3^*J*(^119/117^Sn-^13^C) = 40.2 Hz), 135.1 (^3^*J*(^119/117^Sn-^13^C) = 30.5 Hz), 135.9 (^3^*J*(^119/117^Sn-^13^C) = 29.9 Hz), 140.1, 142.5.

3-(Tributylstannyl)benzyl methanesulfonate (**6**)

Under argon atmosphere, to a solution of 3-(tributylstannyl)benzyl alcohol (**5**) (7.12 g, 17.9 mmol) in CH_2_Cl_2_ (35 mL) were successively added triethylamine (3.80 mL, 27.3 mmol) and methanesulfonyl chloride (1.66 mL, 21.5 mmol) at 0 °C and the mixture was stirred at the same temperature for 3 h. To this was added water (100 mL) and the mixture was extracted with CH_2_Cl_2_ (80 mL × 3). The combined organic extracts were successively washed with water (50 mL × 1) and brine (50 mL × 1), dried (Na_2_SO_4_), filtered, and concentrated under reduced pressure. The residue was purified by flash column chromatography (silica-gel 220 g, *n*-hexane/EtOAc = 9/1) to give 3-(tributylstannyl)benzyl methanesulfonate (**6**) (4.85 g, 56.9%) as a colorless oil; TLC 0.48 (*n*-hexane/EtOAc = 4/1), *R*_f_ = 0.65 (*n*-hexane/CH_2_Cl_2_/EtOAc = 5/2/1); ^1^H NMR (500 MHz, CDCl_3_) δ 0.89 (t, 9H, *J* = 14.6 Hz, 3CH_3_), 0.90–1.14 (m, 6H, 3CH_2_), 1.29–1.37 (m, 6H, 3CH_2_), 1.48–1.60 (m, 6H, 3CH_2_), 2.88 (s, 3H, CH_3_), 5.24 (s, 2H, benzylic CH_2_), 7.31–7.49 (m, 4H, aromatic); ^13^C NMR (125 MHz, CDCl_3_) δ 9.6 (3C, ^1^*J*(^119^Sn-^13^C) = 340.1 Hz, ^1^*J*(^117^Sn-^13^C) = 324.6 Hz), 13.7 (3C), 27.3 (3C, ^1^*J*(^119^Sn-^13^C) = 68.8 Hz, ^1^*J*(^117^Sn-^13^C) = 38.9 Hz), 29.1 (3C, ^2^*J*(^119/117^Sn-^13^C) = 20.1 Hz), 38.4, 72.0, 128.6 (^3^*J*(^119/117^Sn-^13^C) = 77.2 Hz), 128.9, 132.6, 136.8 (^2^*J*(^119/117^Sn-^13^C) = 30.9 Hz), 137.5 (^2^*J*(^119/117^Sn-^13^C) = 29.0 Hz), 143.3; IR (KBr, cm^–1^) 507, 529, 700, 783, 829, 918, 935, 1175, 1356, 1464, 2851, 2870, 2926, 2957; Anal. Calcd. for C_20_H_36_O_3_SSn: C, 50.54; H, 7.63. Found: C, 50.32; H, 7.28; HRMS (FAB^+^/NBA+NaI) *m*/*z* 499.1322 (M+H, C_20_H_36_O_3_S^120^SnNa requires 499.1305).

3-(Tributylstannyl)benzyl bromide (**7**)

Under argon atmosphere, to a solution of 3-(tributylstannyl)benzyl methanesulfonate (**6**) (4.46 g, 9.38 mmol) in DMF (30 mL) was added sodium bromide (1.93 g, 18.8 mmol) at room temperature and the mixture was stirred at the same temperature for 16 h. To this was added water (200 mL) and the mixture was extracted with Et_2_O (80 mL × 3). The combined organic extracts were successively washed with water (50 mL × 3) and brine (50 mL × 1), dried (Na_2_SO_4_), filtered, and concentrated under reduced pressure. The residue was purified by flash column chromatography (silica-gel 100 g, *n*-hexane/Et_2_O = 20/1) to give 3-(tributylstannyl)benzyl bromide (**7**) (4.03 g, 93.3%) as a colorless oil; TLC *R*_f_ = 0.68 (*n*-hexane); ^1^H NMR (500 MHz, CDCl_3_) δ 0.89 (t, 9H, *J* = 7.3 Hz, 3CH_3_), 0.99–1.13 (m, 6H, 3CH_2_), 1.29–1.37 (m, 6H, 3CH_2_), 1.47–1.60 (m, 6H, 3CH_2_), 4.49 (s, 2H, benzylic CH_2_), 7.27–7.49 (m, 4H, aromatic); ^13^C NMR (125 MHz, CDCl_3_) δ 9.6 (3C, ^1^*J*(^119^Sn-^13^C) = 336.9 Hz, ^1^*J*(^117^Sn-^13^C) = 323.8 Hz), 13.7 (3C), 27.3 (3C, ^3^*J*(^119/117^Sn-^13^C) = 55.7 Hz), 29.0 (3C, ^2^*J*(^119/117^Sn-^13^C) = 20.1 Hz), 34.1, 128.1 (^3^*J*(^119/117^Sn-^13^C) = 39.9 Hz), 128.7, 136.5 (^2^*J*(^119/117^Sn-^13^C) = 28.9 Hz), 136.9, 137.1, 142.8; IR (KBr, cm^–1^) 664, 700, 1125, 1462, 2851, 2870, 2924, 2955; Anal. Calcd. for C_19_H_33_BrSn: C, 49.60; H, 7.23. Found: C, 49.66; H, 6.84.

4-((4*H*-1,2,4-Triazol-4-yl)(3-(tributylstannyl)benzyl)amino)benzonitrile (**3**, precursor of [^11^C]meta-cetrozole)

Under argon atmosphere, a mixture of 4-(4*H*-1,2,4-triazol-4-ylamino)benzonitrile (**1**) (500 mg, 2.70 mmol), 3-(tributylstannyl)benzyl bromide (**7**) (1.49 g, 3.24 mmol), and potassium carbonate (746 mg, 5.40 mmol) in acetone (30 mL) was stirred at room temperature for 21 h. The mixture was concentrated under reduced pressure. To the residue was added water (100 mL) and the mixture was extracted with CH_2_Cl_2_ (70 mL × 3). The combined organic extracts were washed with water (50 mL × 1), dried (Na_2_SO_4_), filtered, and concentrated under reduced pressure. The residue was purified by flash column chromatography (silica-gel 60 g, CH_2_Cl_2_ to CH_2_Cl_2_/CH_3_OH = 40/1) to give 4-((4*H*-1,2,4-triazol-4-yl)(3-(tributylstannyl)benzyl)amino)-benzonitrile (**3**, precursor of [^11^C]meta-cetrozole) (1.46 g, 95.8%) as a colorless solid. Recrystallization from *n*-hexane (30 mL) afforded colorless plates (1.13 g, 74.2%); TLC *R*_f_ = 0.63 (CH_2_Cl_2_/EtOAc = 9/1); Mp 116–117 ˚C; ^1^H NMR (500 MHz, CDCl_3_) δ 0.87 (t, 9H, *J* = 7.3 Hz, 3CH_3_), 0.96–1.10 (m, 6H, 3CH_2_), 1.26–1.34 (m, 6H, 3CH_2_), 1.44–1.54 (m, 6H, 3CH_2_), 4.89 (s, 2H, benzylic CH_2_), 6.66–6.68 (AA’BB’, 2H, aromatic), 7.09 (d, 1H, *J* = 7.7 Hz, aromatic), 7.26–7.31 (m, 2H, aromotic), 7.44–7.45 (m, 1H, aromatic), 7.58–7.59 (AA’BB’, 2H, aromatic), 8.08 (s, 2H, triazole); ^13^C NMR (125 MHz, CDCl_3_) δ 9.6 (3C, ^1^*J*(^119^Sn-^13^C) = 340.4 Hz, ^1^*J*(^117^Sn-^13^C) = 324.8 Hz), 13.7 (3C), 27.3 (3C, ^3^*J*(^119/117^Sn-^13^C) = 55.9 Hz), 29.0 (3C, ^2^*J*(^119/117^Sn-^13^C) = 20.2 Hz), 58.4, 105.0, 113.3 (2C), 118.5, 127.6, 128.7, 132.8, 134.1 (2C), 135.7, 137.2, 142.6 (2C), 144.1, 150.5; IR (KBr, cm^–1^) 704, 837, 874, 1069, 1179, 1269, 1379, 1462, 1508, 1605, 2224, 2851, 2870, 2924, 2957; Anal. Calcd. for C_28_H_39_N_5_Sn: C, 59.59; H, 6.97; N, 12.41. Found: C, 59.55; H, 6.81; N, 12.38.

*Synthesis of 4-((4-methylbenzyl)(4H-1,2,4-triazol-4-yl)amino)nitrobenzene (****11****, nitro-cetrozole) and 4-((4H-1,2,4-triazol-4-yl)(4-(tributylstannyl)benzyl)amino)nitrobenzene (****12****, precursor of [^11^C]nitro-cetrozole)*

**Supplemental Fig S2.** Synthetic scheme of nitro-cetrozole (**11**) and precursor of [^11^C]nitro-cetrozole.

4-(4*H*-1,2,4-Triazol-4-ylamino)nitrobenzene (**10**)^4^

Under argon atmosphere, to a solution of potassium *tert*-butoxide (16.3 g, 145 mmol) in DMSO (80 mL) was added 4-amino-4*H*-1,2,4-triazole (**9**) (12.2 g, 145 mmol) at 0 °C and the mixture was warmed to room temperature. After stirring for 5 min, to this was added a solution of 4-fluoronitrobenzene (**8**) (7.70 mL, 72.5 mmol) in DMSO (10 mL) keeping the temperature of the reaction mixture below 30 °C. After stirring for 1 h at room temperature, the mixture was poured into a 2 M aqueous solution of HCl and neutralized to pH 7 by adding an aqueous solution saturated with NaHCO_3_. The volume of the mixture was increased to 1 L by adding water and then cooled at 4 °C for 2 h. The resulting precipitate was collected by filtration, washed with water on a funnel, and dried under reduced pressure. Recrystallization from DMSO (100 mL) and water (100 mL) gave 4-(4*H*-1,2,4-triazol-4-ylamino)nitrobenzene (**10**) (4.29 g, 28.8%) as brown plates; TLC *R*_f_ = 0.36 (CH_2_Cl_2_/CH_3_OH = 7/1); ^1^H NMR (500 MHz, DMSO-*d*_6_) δ 6.57–6.59 (AA’BB’, 2H, aromatic), 8.14–8.16 (AA’BB’, 2H, aromatic), 8.87 (s, 2H, triazole), 10.51 (s, 1H, NH); ^13^C NMR (125 MHz, DMSO-*d*_6_) δ 111.6 (2C), 126.1 (2C), 140.3, 144.0 (2C), 152.7.

4-((4-Methylbenzyl)(4*H*-1,2,4-triazol-4-yl)amino)nitrobenzene (**11**, nitro-cetrozole)^1^

Under argon atmosphere, a mixture of 4-(4*H*-1,2,4-triazol-4-ylamino)nitrobenzene (**10**) (500 mg, 2.44 mmol), 4-methylbenzyl bromide (542 mg, 2.93 mmol), and potassium carbonate (674 mg, 4.88 mmol) in acetone (15 mL) was stirred at room temperature for 22 h. To this was added water (100 mL) and the mixture was extracted with EtOAc (100 mL × 3). The combined organic extracts were successively washed with water (50 mL × 1) and brine (50 mL × 1), dried (Na_2_SO_4_), filtered, and concentrated under reduced pressure. The residue was purified by flash column chromatography (silica-gel 60 g, *n*-hexane/EtOAc = 1/2) to give 4-((4-methylbenzyl)(4*H*-1,2,4-triazol-4-yl)amino)nitrobenzene (**11**, nitro-cetrozole) (340 mg, 45.1%) as a slightly brown solid. Recrystallization from EtOAc (60 mL) and *n*-hexane (30 mL) afforded slightly yellow plates (254 mg, 33.7%); TLC *R*_f_ = 0.48 (*n*-hexane/EtOAc = 1/4); ^1^H NMR (500 MHz, DMSO-*d*_6_) δ 2.26 (s, 3H, CH_3_), 5.06 (s, 2H, benzylic CH_2_), 6.77–6.79 (AA’BB’, 2H, aromatic), 7.11–7.13 (AA’BB’, 2H, aromatic), 7.18–7.19 (AA’BB’, 2H, aromatic), 8.17–8.19 (AA’BB’, 2H, aromatic), 8.78 (s, 2H, triazole); ^13^C NMR (125 MHz, DMSO-*d*_6_) δ 20.7, 57.0, 113.0 (2C), 125.8 (2C), 128.4 (2C), 129.2 (2C), 131.3, 137.4, 140.6, 143.3 (2C), 153.1; IR (KBr, cm^–1^) 665, 748, 818, 835, 851, 870, 1063, 1111, 1188, 1211, 1223, 1288, 1335, 1395, 1495, 1595, 3067, 3123; Anal. Calcd. for C_16_H_15_N_5_O_2_: C, 62.13; H, 4.89; N, 22.64. Found: C, 62.21; H, 4.88; N, 22.68.

4-((4*H*-1,2,4-Triazol-4-yl)(4-(tributylstannyl)benzyl)amino)nitrobenzene (**12**, precursor of [^11^C]nitro-cetrozole)

Under argon atmosphere, a mixture of 4-(4*H*-1,2,4-triazol-4-ylamino)nitrobenzene (**10**) (449 mg, 2.19 mmol), 4-(tributylstannyl)benzyl bromide (1.21 g, 2.63 mmol), and potassium carbonate (605 mg, 4.38 mmol) in acetone (20 mL) was stirred at room temperature for 18 h. To this was added water (100 mL) and the mixture was extracted with EtOAc (80 mL × 3). The combined organic extracts were successively washed with water (50 mL × 1) and brine (50 mL × 1), dried (Na_2_SO_4_), filtered, and concentrated under reduced pressure. The residue was purified by flash column chromatography (silica-gel 60 g, *n*-hexane/EtOAc = 1/1) to give 4-((4*H*-1,2,4-triazol-4-yl)(4-(tributylstannyl)benzyl)amino)nitrobenzene (**12**, precursor of [^11^C]nitro-cetrozole) (718 mg, 56.1%) as a slightly brown solid. Recrystallization from EtOAc (3 mL) and *n*-hexane (30 mL) afforded slightly yellow plates (534 mg, 41.8%); TLC *R*_f_ = 0.43 (*n*-hexane/EtOAc = 1/1); Mp 112–113 ˚C; ^1^H NMR (500 MHz, CDCl_3_) δ 0.88 (t, 9H, *J* = 7.3 Hz, 3CH_3_), 0.99–1.12 (m, 6H, 3CH_2_), 1.28–1.36 (m, 6H, 3CH_2_), 1.49–1.55 (m, 6H, 3CH_2_), 4.94 (s, 2H, benzylic CH_2_), 6.64–6.68 (AA’BB’, 2H, aromatic), 7.14–7.16 (AA’BB’, 2H, aromatic), 7.41–7.50 (AA’BB’, 2H, ^1^*J*(^119^Sn-^1^H) = 43.7 Hz, ^1^*J*(^117^Sn-^1^H) = 28.5 Hz), aromatic), 8.14 (s, 2H, triazole), 8.18–8.20 (AA’BB’, 2H, aromatic); ^13^C NMR (125 MHz, CDCl_3_) δ 9.6 (3C, ^1^*J*(^119^Sn-^13^C) = 340.0 Hz, ^1^*J*(^117^Sn-^13^C) = 325.3 Hz), 13.6 (3C), 27.3 (3C, ^3^*J*(^119/117^Sn-^13^C) = 55.7 Hz), 29.0 (3C, ^2^*J*(^119/117^Sn-^13^C) = 20.1 Hz), 58.4, 112.4 (2C), 126.1 (2C), 127.3 (2C, ^3^*J*(^119/117^Sn-^13^C) = 39.3 Hz), 132.8, 137.4 (2C, ^2^*J*(^119/117^Sn-^13^C) = 30.3 Hz), 141.9, 142.6 (2C), 144.0, 151.9; IR (KBr, cm^–1^) 596, 617, 637, 665, 691, 750, 837, 860, 997, 1016, 1065, 1123, 1132, 1196, 1221, 1263, 1339, 1375, 1395, 1460, 1501, 1595, 2851, 2924, 3117; Anal. Calcd. for C_27_H_39_N_5_O_2_Sn: C, 55.50; H, 6.73; N, 11.99. Found: C, 55.48; H, 6.36; N, 11.95.

*Synthesis of 4-((4-methylbenzyl)(1H-1,2,4-triazol-1-yl)amino)benzonitrile (****16****, iso-cetrozole) and 4-((1H-1,2,4-triazol-1-yl)(4-(tributylstannyl)benzyl)amino)benzonitrile (****17****, precursor of [^11^C]iso-cetrozole)*

**Supplemental Fig S3.** Synthetic scheme of iso-cetrozole (**16**) and precursor of [^11^C]iso-cetrozole.

1-Amino-1*H*-1,2,4-triazole (**14**)^5^

To a solution of potassium hydroxide (40.7 g, 500 mmol) in EtOH (40 mL) was slowly added 1,2,4-triazole (**18**) (6.91 g, 100 mmol) at 0 °C. To this was added a solution of hydroxylamine-*O*-sulfonic acid (22.6 g, 200 mmol) in H_2_O–EtOH (20 mL–20 mL) at 0 °C and the mixture was allowed to warm to room temperature. After stirring for 2 h, the resulting precipitate was removed by filtration and the filtrate was concentrated under reduced pressure to remove EtOH. To the residue was added THF (400 mL) and after stirring the mixture at room temperature for 30 min, the THF layer was separated and concentrated under reduced pressure. To the residue was added Et_2_O (400 mL) and after stirring the mixture at room temperature for 30 min, the Et_2_O layer was separated and concentrated under reduced pressure. The residue was purified by recrystallization from Et_2_O (400 mL) and *n*-hexane (100 mL) to give 1-amino-1*H*-1,2,4-triazole (**14**) (3.69 g, 43.9%) as a colorless solid; TLC *R*_f_ = 0.39 (CH_2_Cl_2_/CH_3_OH = 7/1); ^1^H NMR (500 MHz, DMSO-*d*_6_) δ 6.53 (s, 2H, NH_2_), 7.80 (s, 1H, triazole), 8.29 (s, 1H, triazole); ^13^C NMR (125 MHz, DMSO-*d*_6_) δ 142.3, 148.9.

4-(1*H*-1,2,4-Triazol-1-ylamino)benzonitrile (**15**)^4^

Under argon atmosphere, to a solution of potassium *tert*-butoxide (668 mg, 5.95 mmol) in DMSO (3 mL) was added 1-amino-1*H*-1,2,4-triazole (**14**) (500 mg, 5.95 mmol) at 0 °C and the mixture was warm to room temperature. After stirring for 10 min, to this was added a solution of 4-fluorobenzonitrile (**13**) (361 mg, 2.98 mmol) in DMSO (1 mL) keeping the temperature of the reaction mixture below 30 °C. After stirring for 1 h at room temperature, the mixture was poured into a 2 M aqueous solution of HCl and neutralized to pH 7 by adding an aqueous solution saturated with NaHCO_3_. The volume of the mixture was increased to 100 mL by adding water and then cooled at 4 °C for 2 h. The resulting precipitate was collected by filtration, washed with water on a funnel, and dried under reduced pressure to give 4-(1*H*-1,2,4-triazol-1-ylamino)benzonitrile (**15**) (360 mg, 65.2%) as a colorless solid; TLC *R*_f_ = 0.46 (CH_2_Cl_2_/CH_3_OH = 7/1); ^1^H NMR (500 MHz, DMSO-*d*_6_) δ 6.52–6.53 (AA’BB’, 2H, aromatic), 7.66–7.68 (AA’BB’, 2H, aromatic), 8.16 (s, 1H, triazole), 8.79 (s, 1H, triazole), 10.51 (s, 1H, NH); ^13^C NMR (125 MHz, DMSO-*d*_6_) δ 102.5, 113.0 (2C), 119.3, 133.9 (2C), 144.8, 150.7, 151.0.

4-((4-Methylbenzyl)(1*H*-1,2,4-triazol-1-yl)amino)benzonitrile (**16**, iso-cetrozole, TMD-322^6^)^4^

Under argon atmosphere, a mixture of 4-(1*H*-1,2,4-triazol-1-ylamino)benzonitrile (**35**) (300 mg, 1.62 mmol), 4-methylbenzyl bromide (359 mg, 1.94 mmol), and potassium carbonate (448 mg, 3.24 mmol) in acetone (15 mL) was stirred at room temperature for 20 h. After concentration of the mixture under reduced pressure, to the residue was added water (50 mL) and the mixture was extracted with CH_2_Cl_2_ (30 mL × 3). The combined organic extracts were successively washed with water (30 mL × 1), dried (Na_2_SO_4_), filtered, and concentrated under reduced pressure. The residue was purified by flash column chromatography (silica-gel 30 g, CH_2_Cl_2_ to CH_2_Cl_2_/EtOAc = 4/1) to give 4-((4-methylbenzyl)(1*H*-1,2,4-triazol-1-yl)amino)benzonitrile (**16**, iso-cetrozole) (448 mg, 95.6%) as a colorless solid. Recrystallization from EtOAc (8 mL) and *n*-hexane (32 mL) afforded colorless plates (395 mg, 84.3%); TLC *R*_f_ = 0.46 (CH_2_Cl_2_/CH_3_OH = 9/1); ^1^H NMR (500 MHz, DMSO-*d*_6_) δ 2.25 (s, 3H, CH_3_), 4.96 (s, 2H, benzylic CH_2_), 6.72–6.74 (AA’BB’, 2H, aromatic), 7.09–7.11 (AA’BB’, 2H, aromatic), 7.19–7.21 (AA’BB’, 2H, aromatic), 7.72–7.73 (AA’BB’, 2H, aromatic), 8.14 (s, 1H, triazole), 8.57 (s, 1H, triazole); ^13^C NMR (125 MHz, DMSO-*d*_6_) δ 20.7, 56.7, 103.3, 114.6 (2C), 119.0, 128.5 (2C), 129.1 (2C), 131.7, 133.7 (2C), 137.2, 145.0, 151.1, 151.4; IR (KBr, cm^–1^) 548, 617, 669, 719, 827, 943, 988, 1022, 1130, 1179, 1204, 1229, 1275, 1304, 1333, 1350, 1368, 1420, 1450, 1510, 1605, 2222, 2864, 2920, 3049, 3123; Anal. Calcd. for C_17_H_15_N_5_: C, 70.57; H, 5.23; N, 24.21. Found: C, 70.76; H, 5.20; N, 24.29.

4-((1*H*-1,2,4-Triazol-1-yl)(4-(tributylstannyl)benzyl)amino)benzonitrile (**17**, precursor of [^11^C]iso-cetrozole)

Under argon atmosphere, a mixture of 4-(1*H*-1,2,4-triazol-1-ylamino)benzonitrile (**15**) (300 mg, 1.62 mmol), 4-(tributylstannyl)benzyl bromide (893 mg, 1.94 mmol), and potassium carbonate (448 mg, 3.24 mmol) in acetone (15 mL) was stirred at room temperature for 21 h. To this was added water (50 mL) and the mixture was extracted with CH_2_Cl_2_ (30 mL × 3). The combined organic extracts were washed with water (30 mL × 1), dried (Na_2_SO_4_), filtered, and concentrated under reduced pressure. The residue was purified by flash column chromatography (silica-gel 60 g, CH_2_Cl_2_ to CH_2_Cl_2_/EtOAc = 4/1) to give 4-((1*H*-1,2,4-triazol-1-yl)(4-(tributylstannyl)benzyl)amino)benzonitrile (**17**, precursor of [^11^C]iso-cetrozole) (891 mg, 97.5%) as a colorless oil; TLC *R*_f_ = 0.63 (CH_2_Cl_2_/EtOAc = 9/1); ^1^H NMR (500 MHz, CDCl_3_) δ 0.88 (t, 9H, *J* = 7.4 Hz, 3CH_3_), 1.03–1.06 (m, 6H, 3CH_2_), 1.28–1.35 (m, 6H, 3CH_2_), 1.49–1.55 (m, 6H, 3CH_2_), 4.89 (s, 2H, benzylic CH_2_), 6.67–6.69 (AA’BB’, 2H, aromatic), 7.19–7.20 (AA’BB’, 2H, aromatic), 7.37–7.46 (AA’BB’, 2H, ^1^*J*(^119^Sn-^1^H) = 44.3 Hz, ^1^*J*(^117^Sn-^1^H) = 29.4 Hz, aromatic), 7.55–7.58 (AA’BB’, 2H, aromatic), 7.86 (s, 1H, triazole), 8.03 (s, 1H, triazole); ^13^C NMR (125 MHz, CDCl_3_) δ 9.6 (3C, ^1^*J*(^119^Sn-^13^C) = 339.5 Hz, ^1^*J*(^117^Sn-^13^C) = 324.5 Hz), 13.6 (3C), 27.3 (3C, ^3^*J*(^119/117^Sn-^13^C) = 56.3 Hz), 29.0 (3C, ^2^*J*(^119/117^Sn-^13^C) = 20.1 Hz), 58.3, 105.3, 114.8 (2C), 118.8, 127.6 (2C, ^3^*J*(^119/117^Sn-^13^C) = 39.8 Hz), 133.5, 133.7 (2C), 137.0 (2C, ^2^*J*(^119/117^Sn-^13^C) = 30.4 Hz), 143.0, 143.9, 151.4, 151.5; IR (KBr, cm^–1^) 669, 826, 986, 1130, 1279, 1273, 1375, 1454, 1508, 1605, 2224, 2851, 2870, 2924, 2955; Anal. Calcd. for C_28_H_39_N_5_Sn: C, 59.59; H, 6.97; N, 12.41. Found: C, 59.41; H, 6.86; N, 12.38.


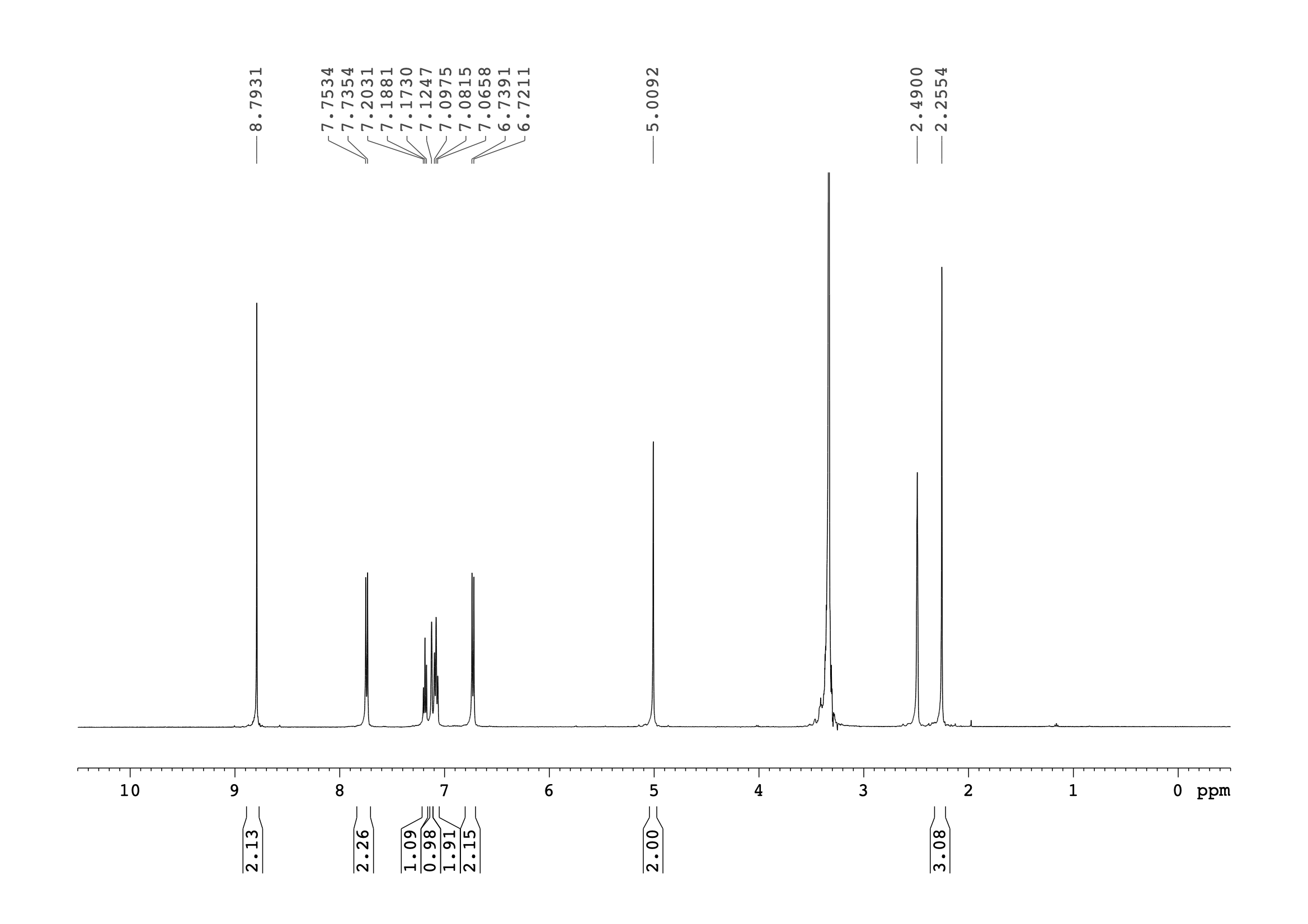


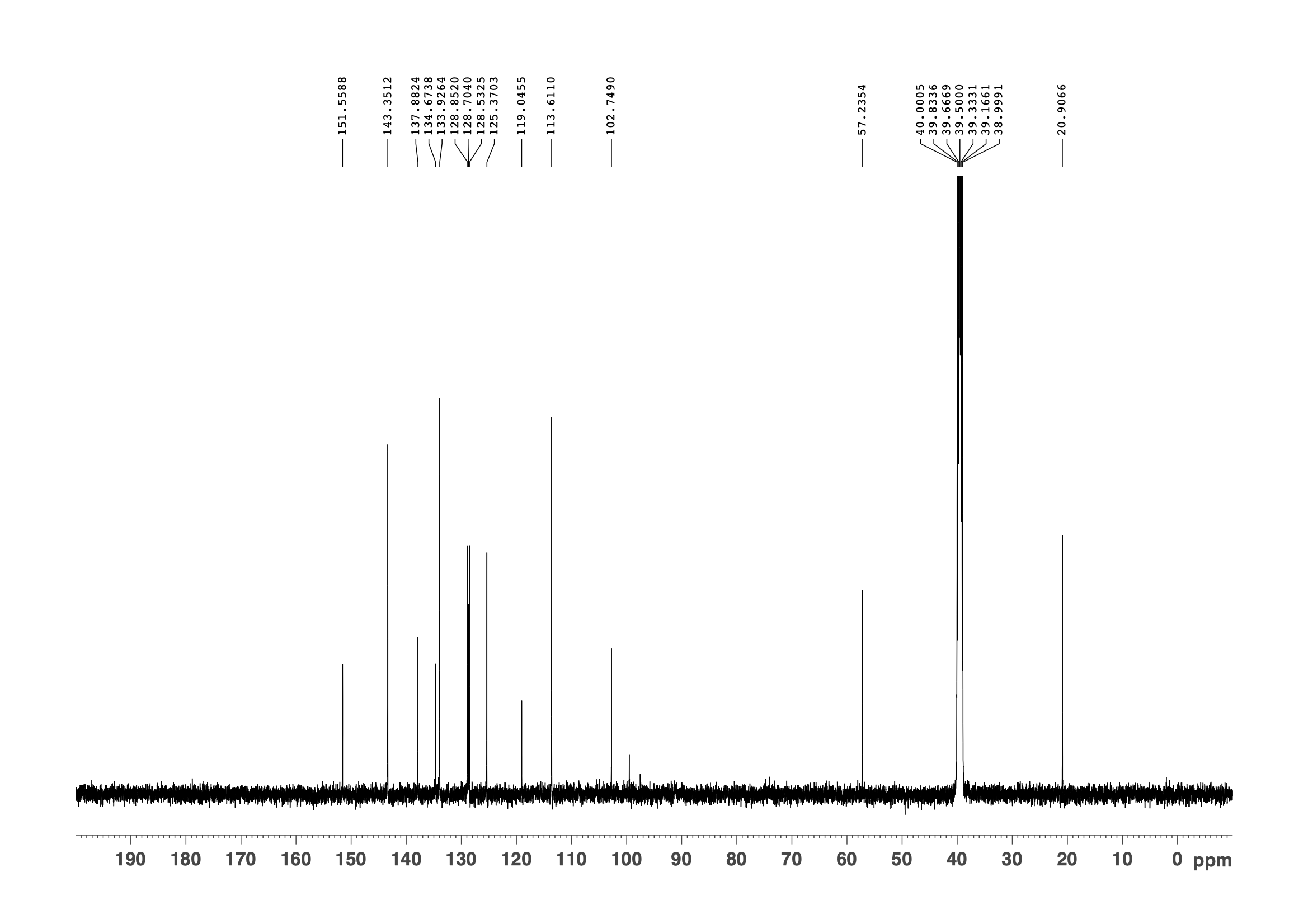


**Supplemental Fig S4.** ^1^H NMR (400 MHz) and ^13^C NMR (100 MHz) spectra of **2** (DMSO-*d*_6_).


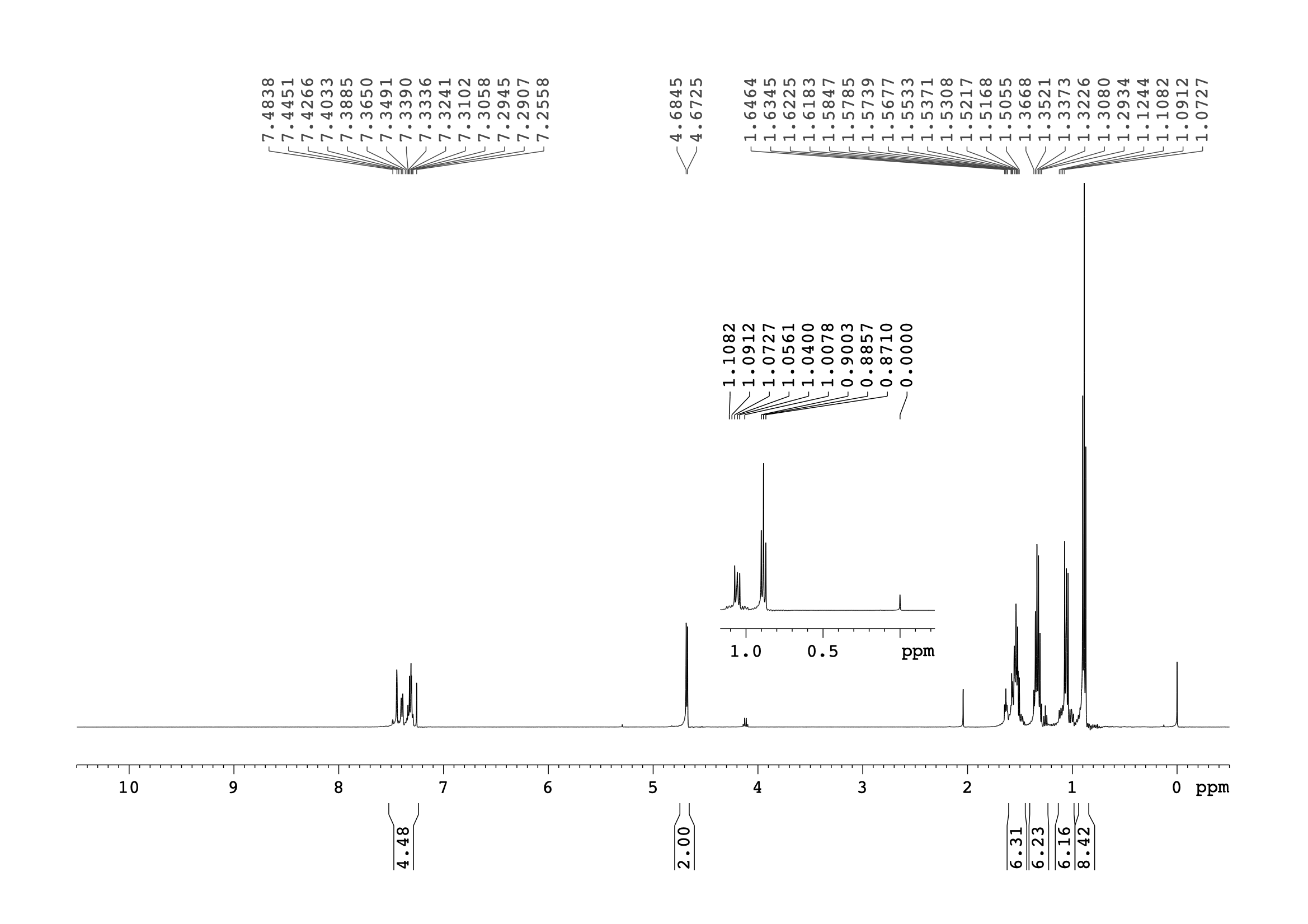


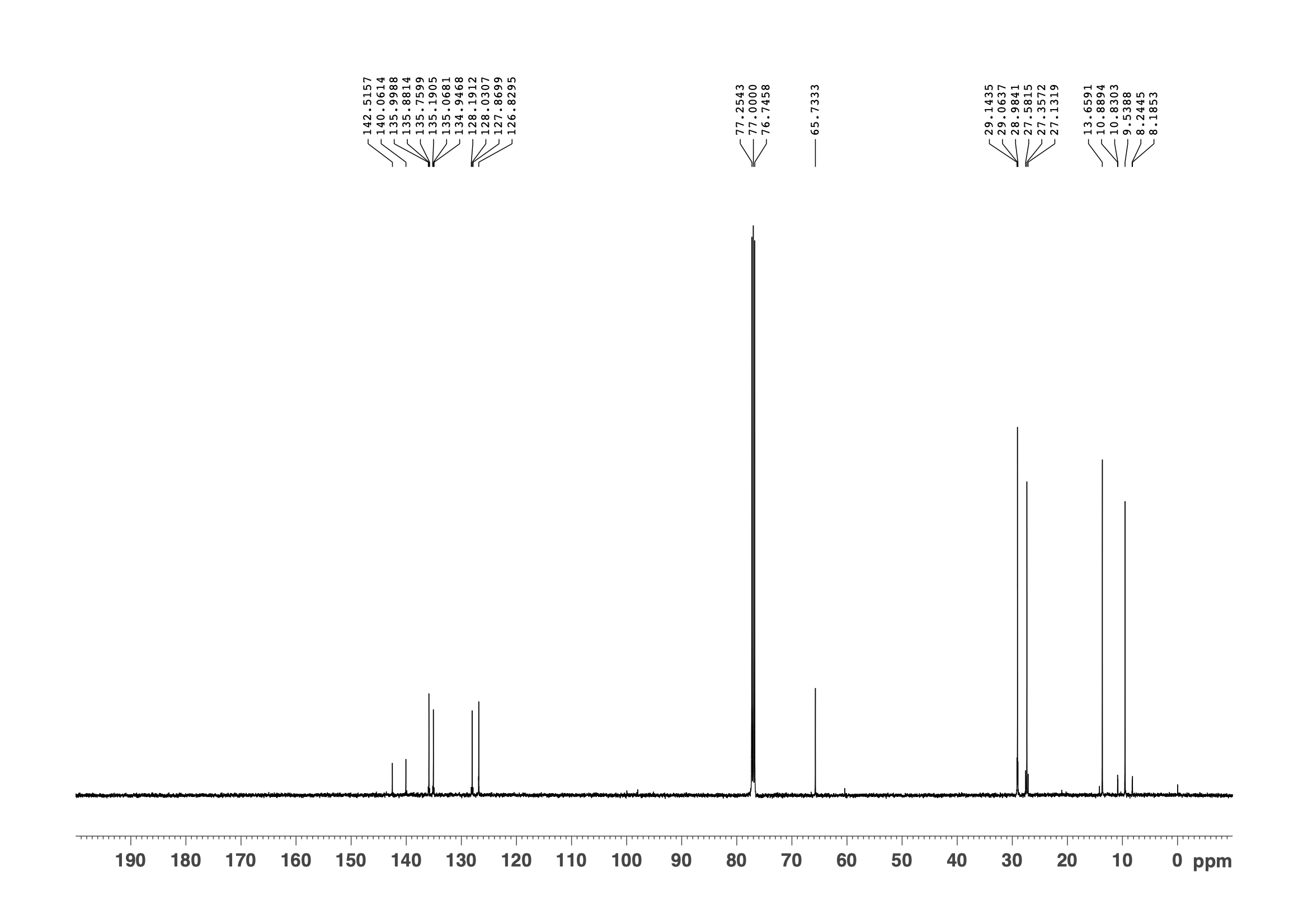


**Supplemental Fig S5.** ^1^H NMR (400 MHz) and ^13^C NMR (100 MHz) spectra of **5** (CDCl_3_).


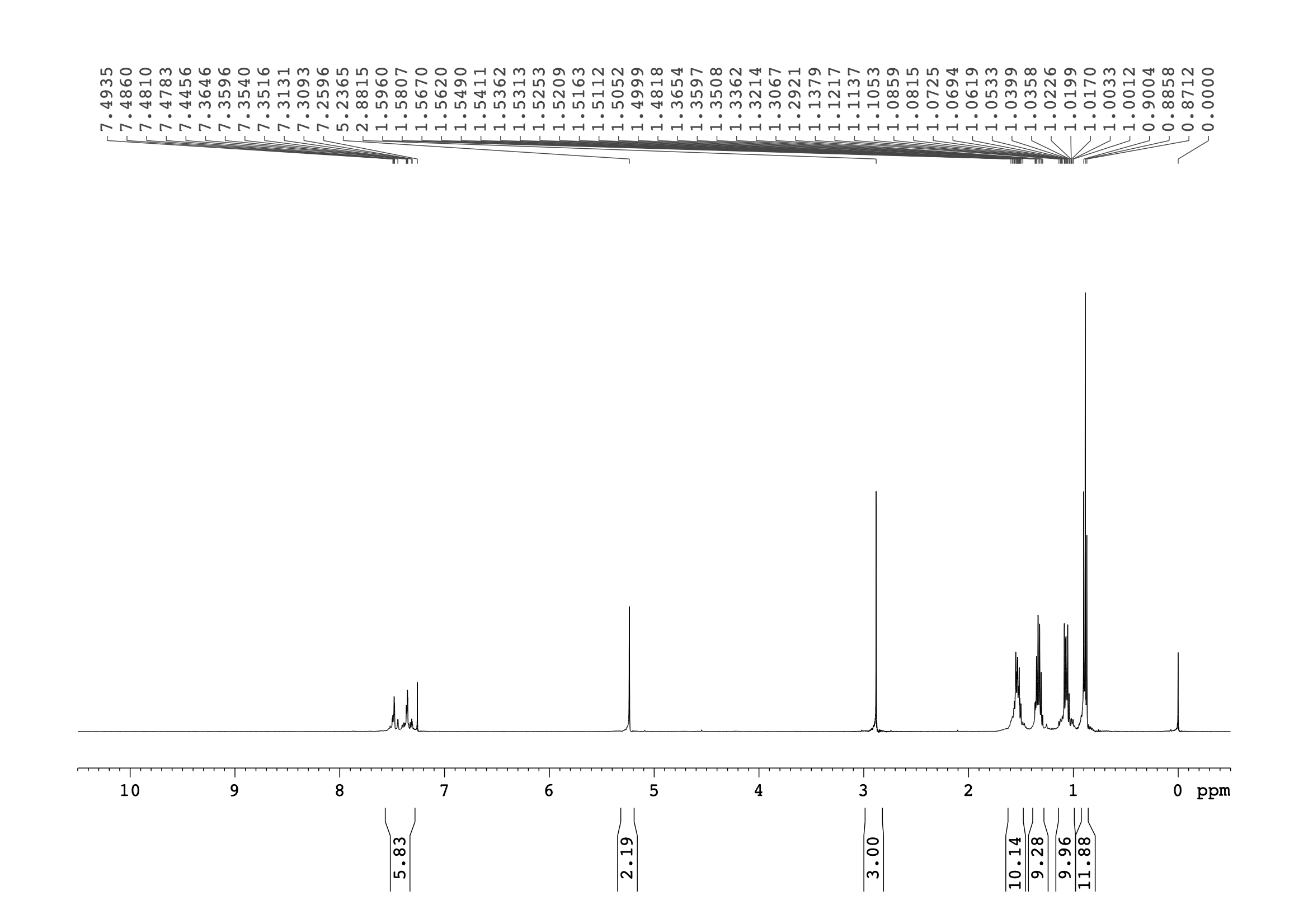


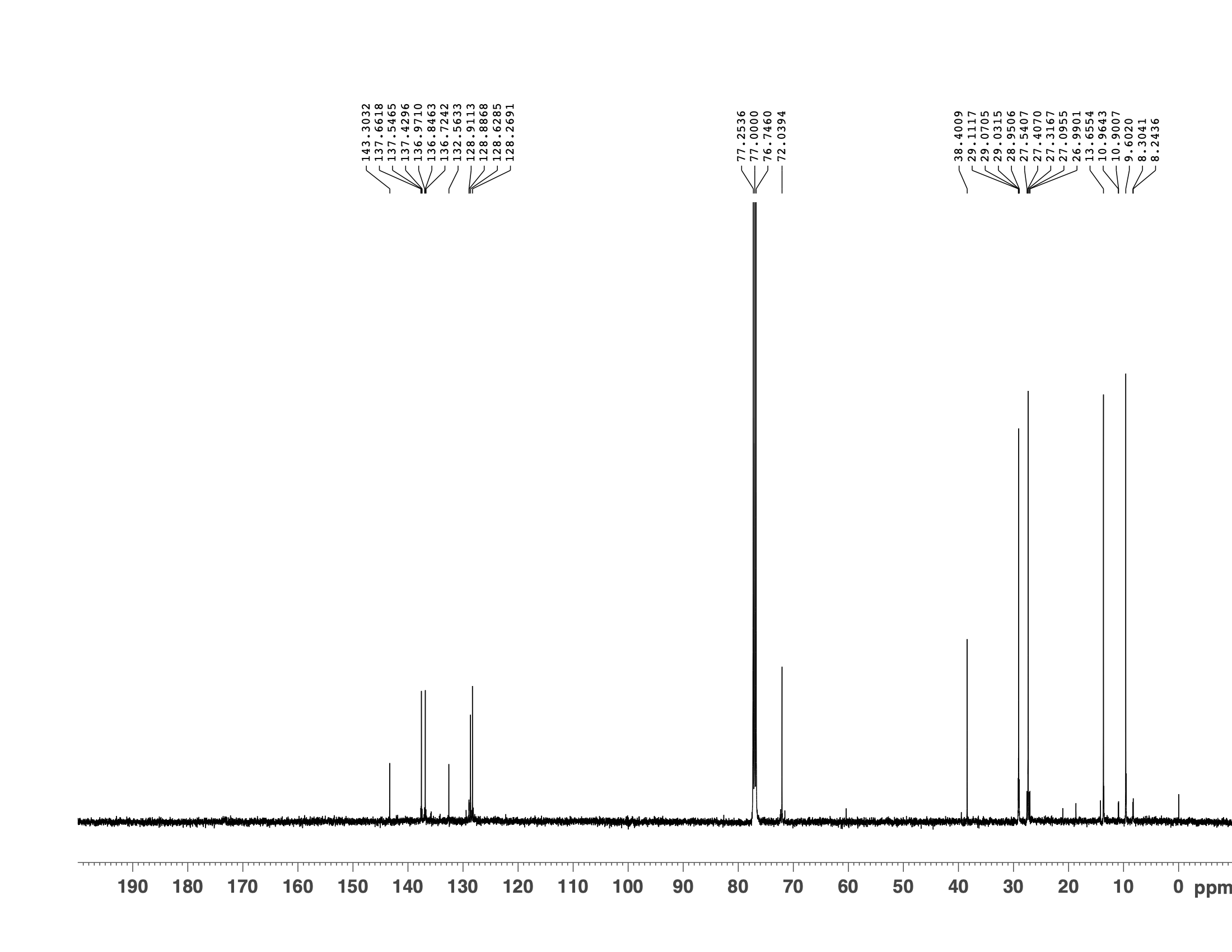


**Supplemental Fig S6.** ^1^H NMR (400 MHz) and ^13^C NMR (100 MHz) spectra of **6** (CDCl_3_).


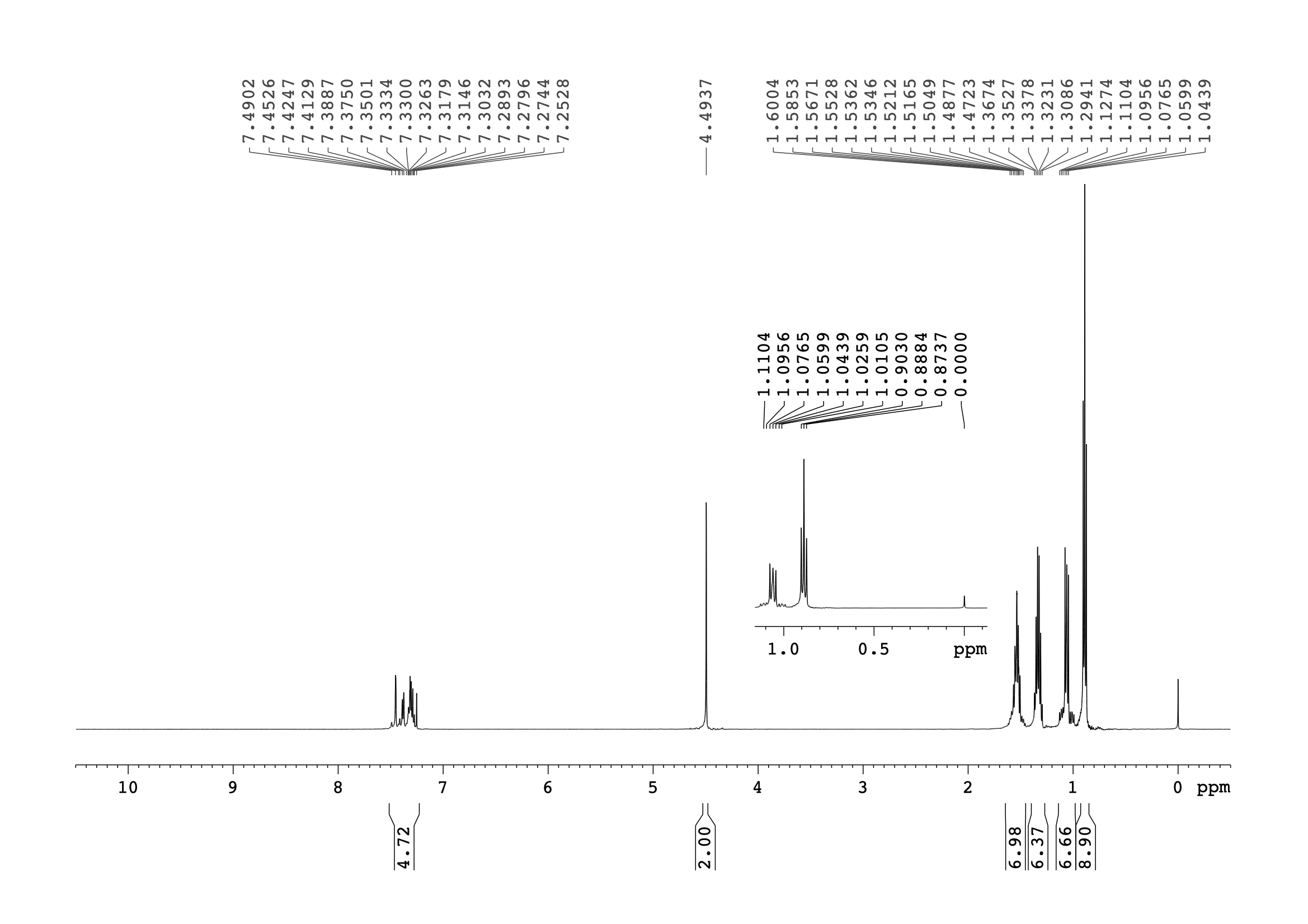


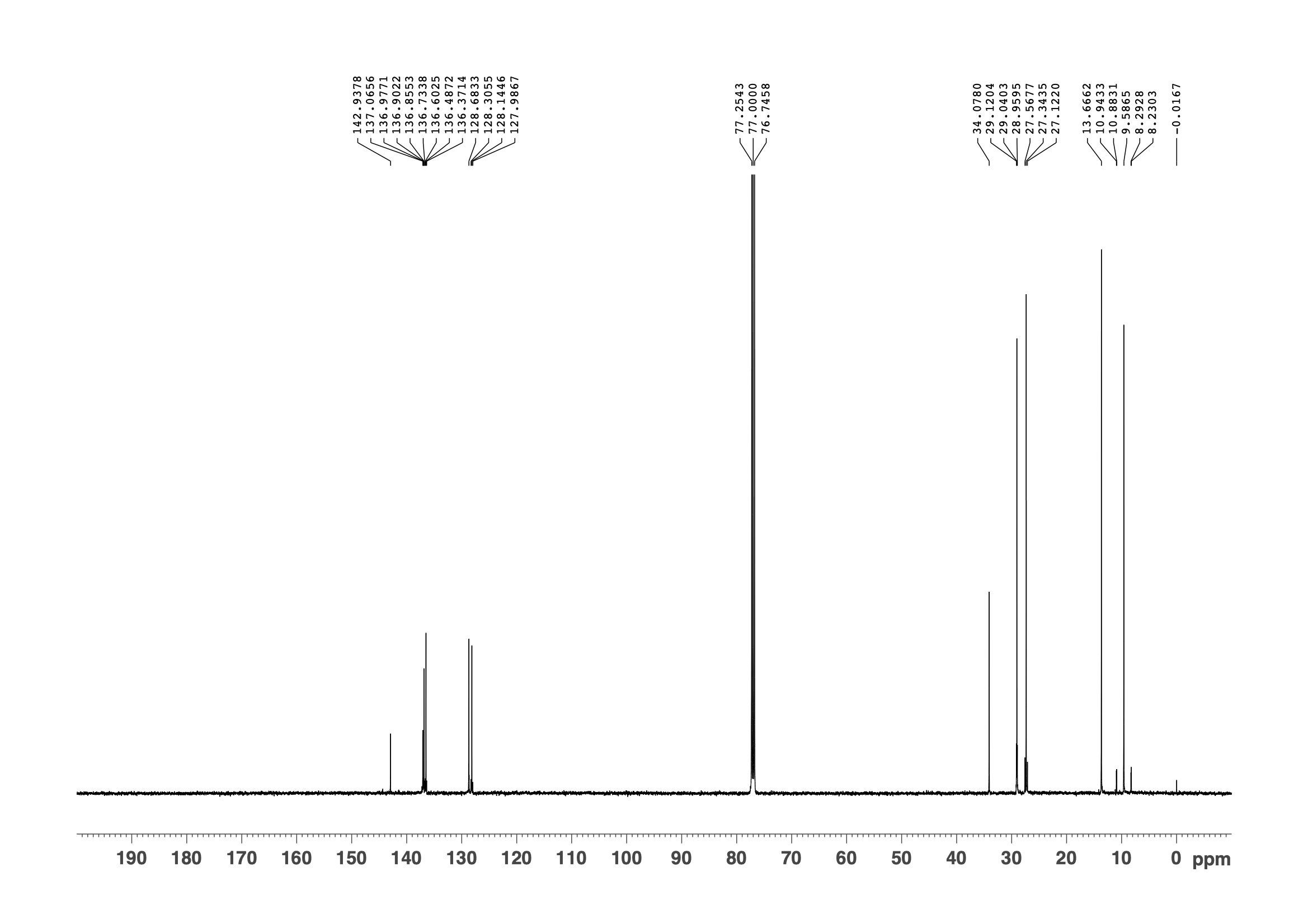


**Supplemental Fig S7.** ^1^H NMR (400 MHz) and ^13^C NMR (100 MHz) spectra of **7** (CDCl_3_).


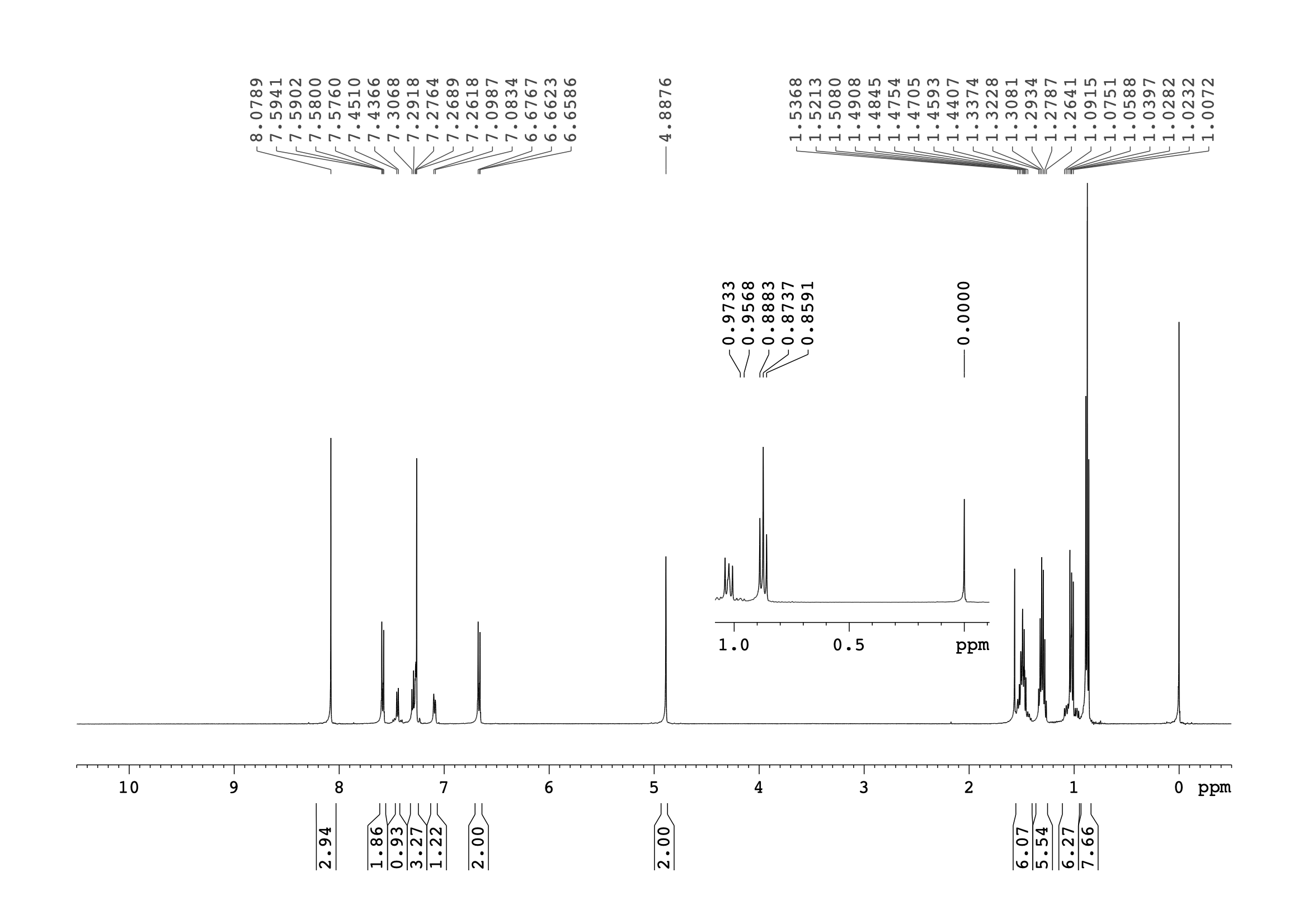


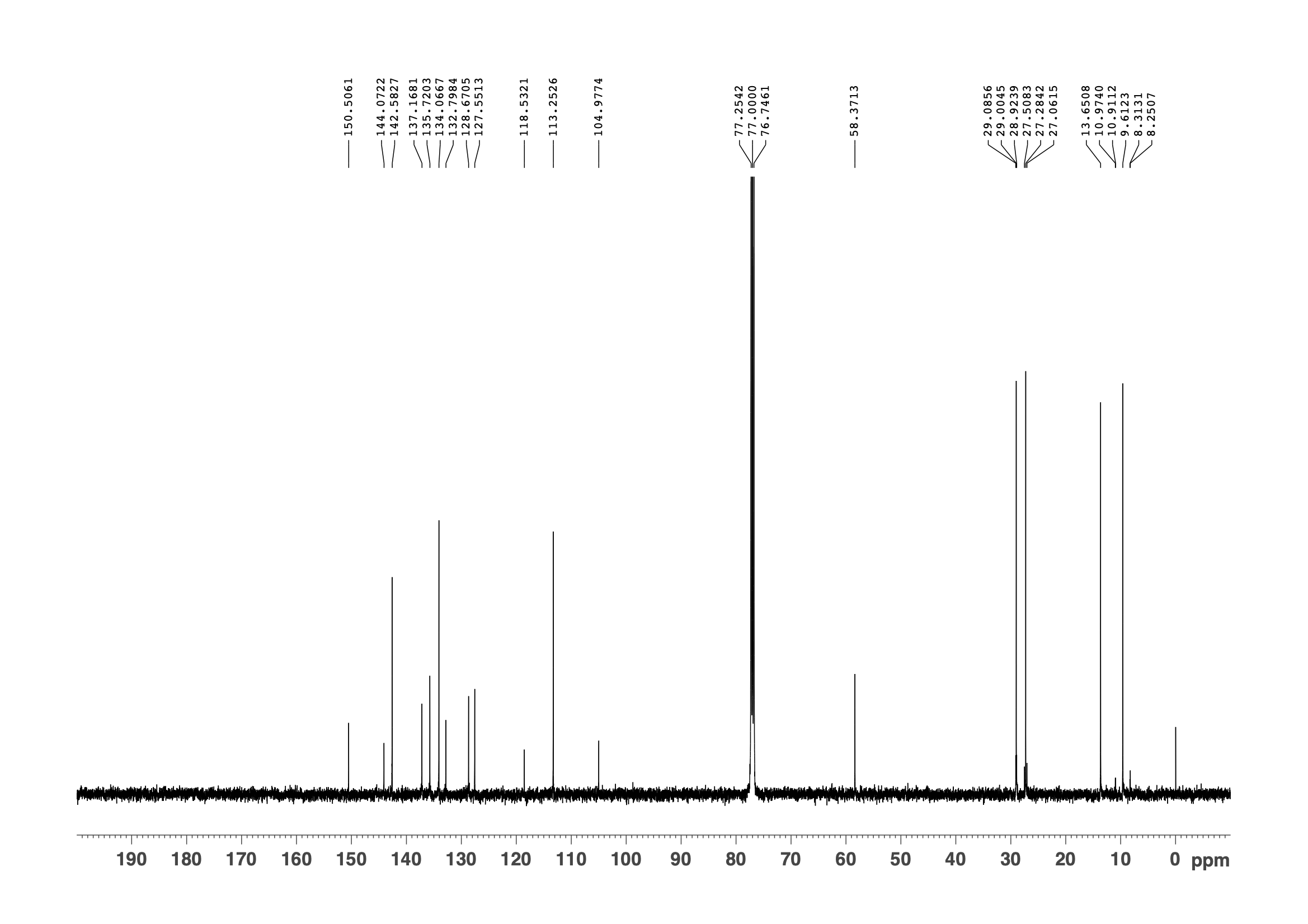


**Supplemental Fig S8.** ^1^H NMR (400 MHz) and ^13^C NMR (100 MHz) spectra of **3** (CDCl_3_).


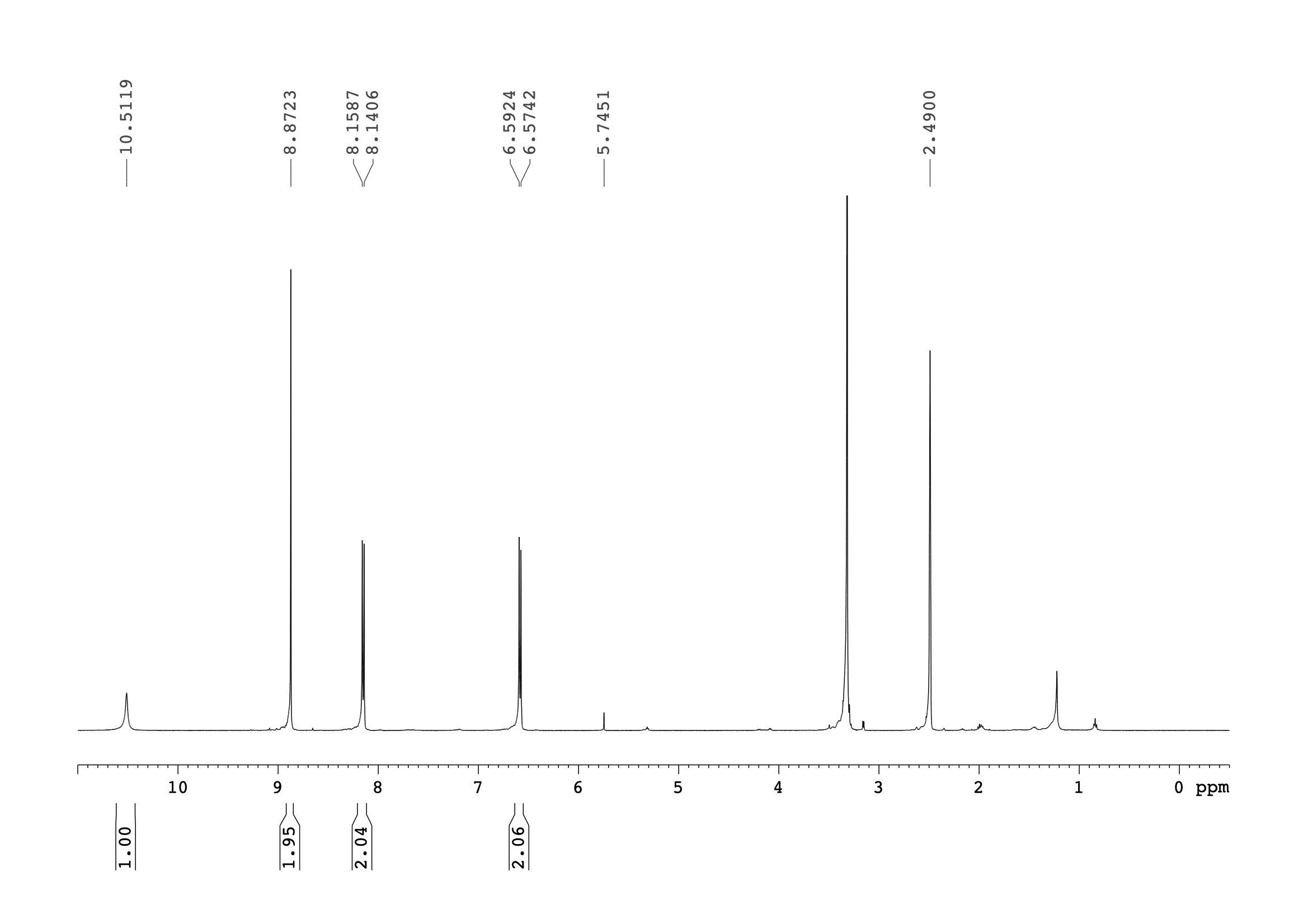


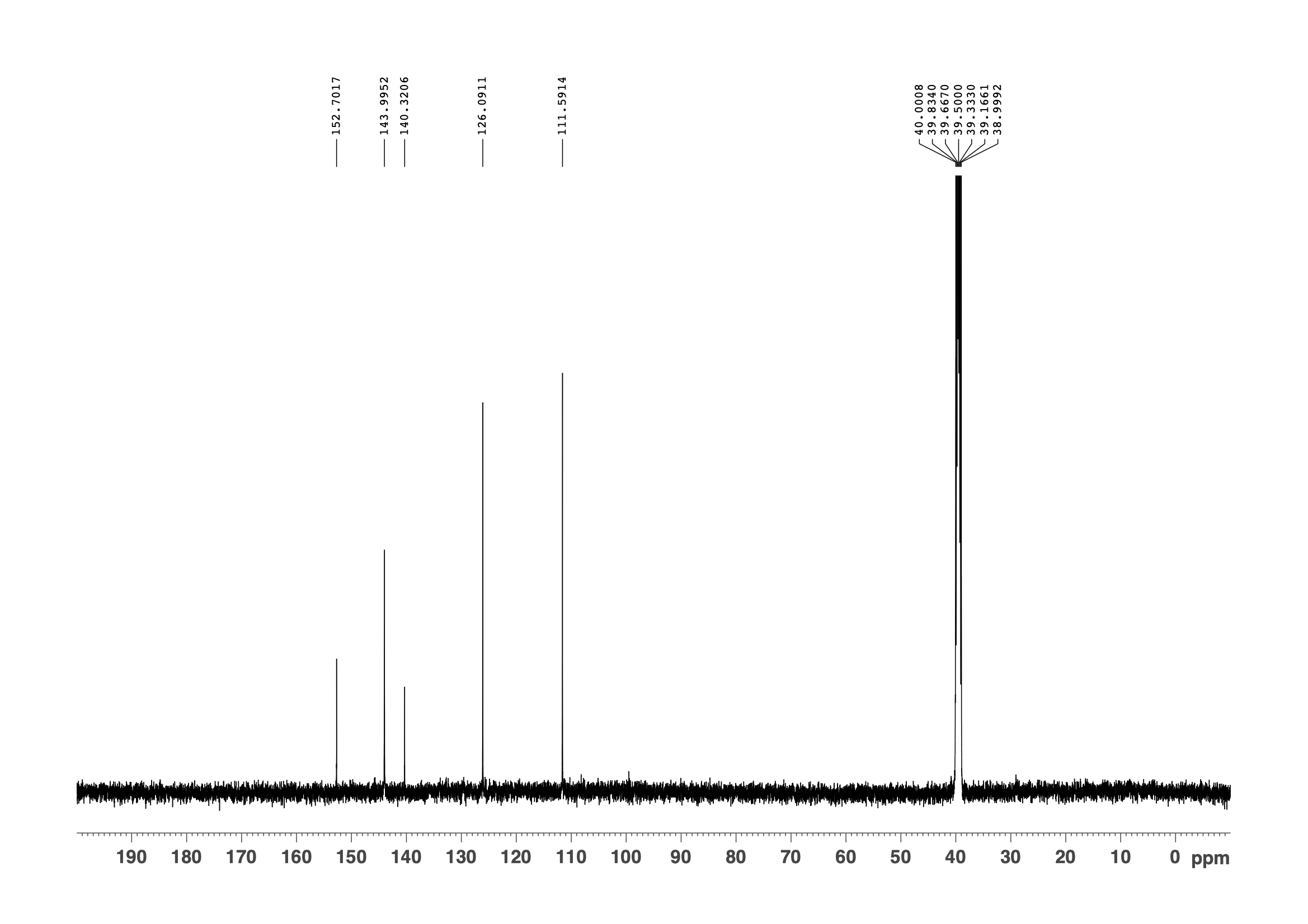


**Supplemental Fig S9.** ^1^H NMR (400 MHz) and ^13^C NMR (100 MHz) spectra of **10** (DMSO-*d*_6_).


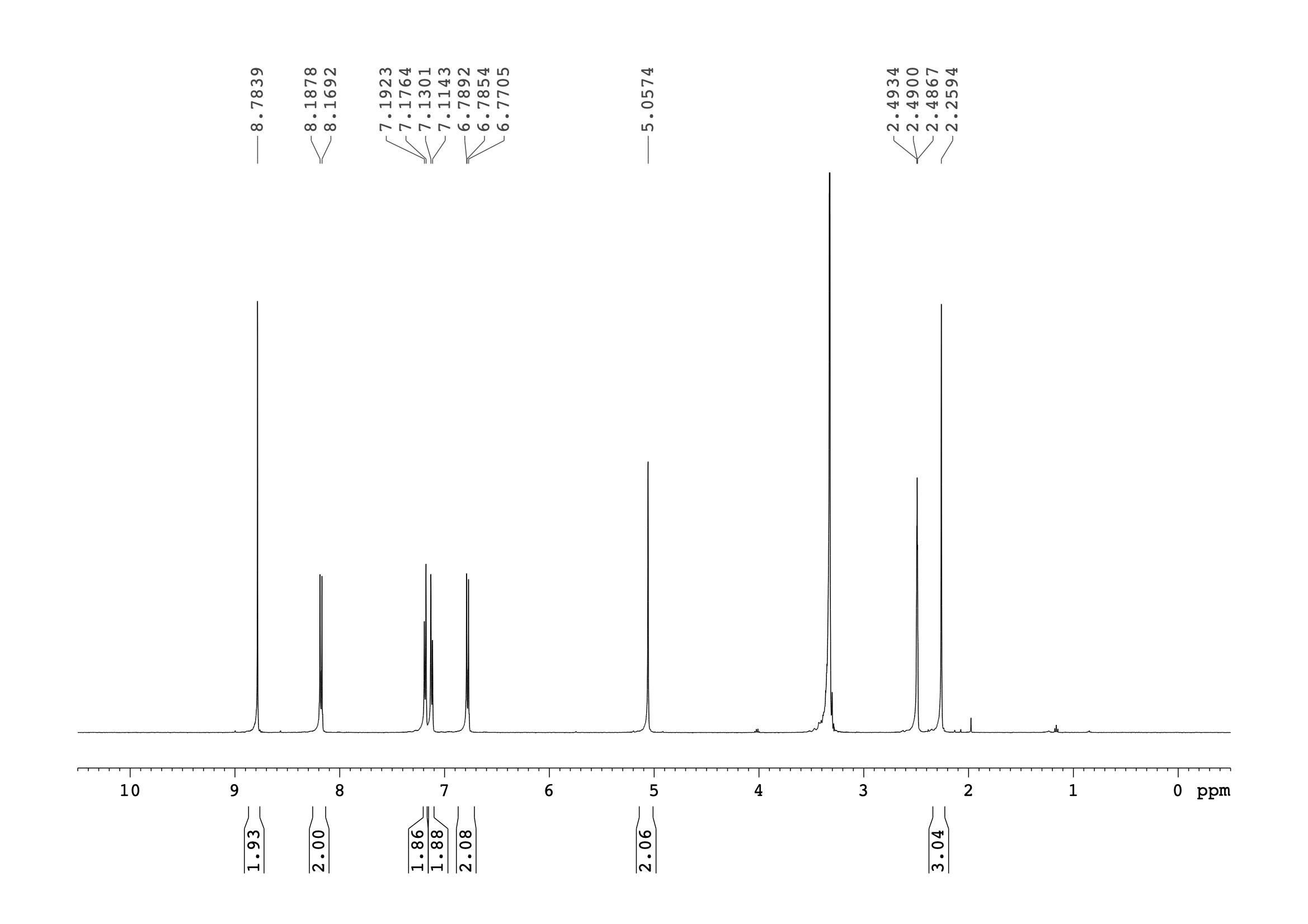


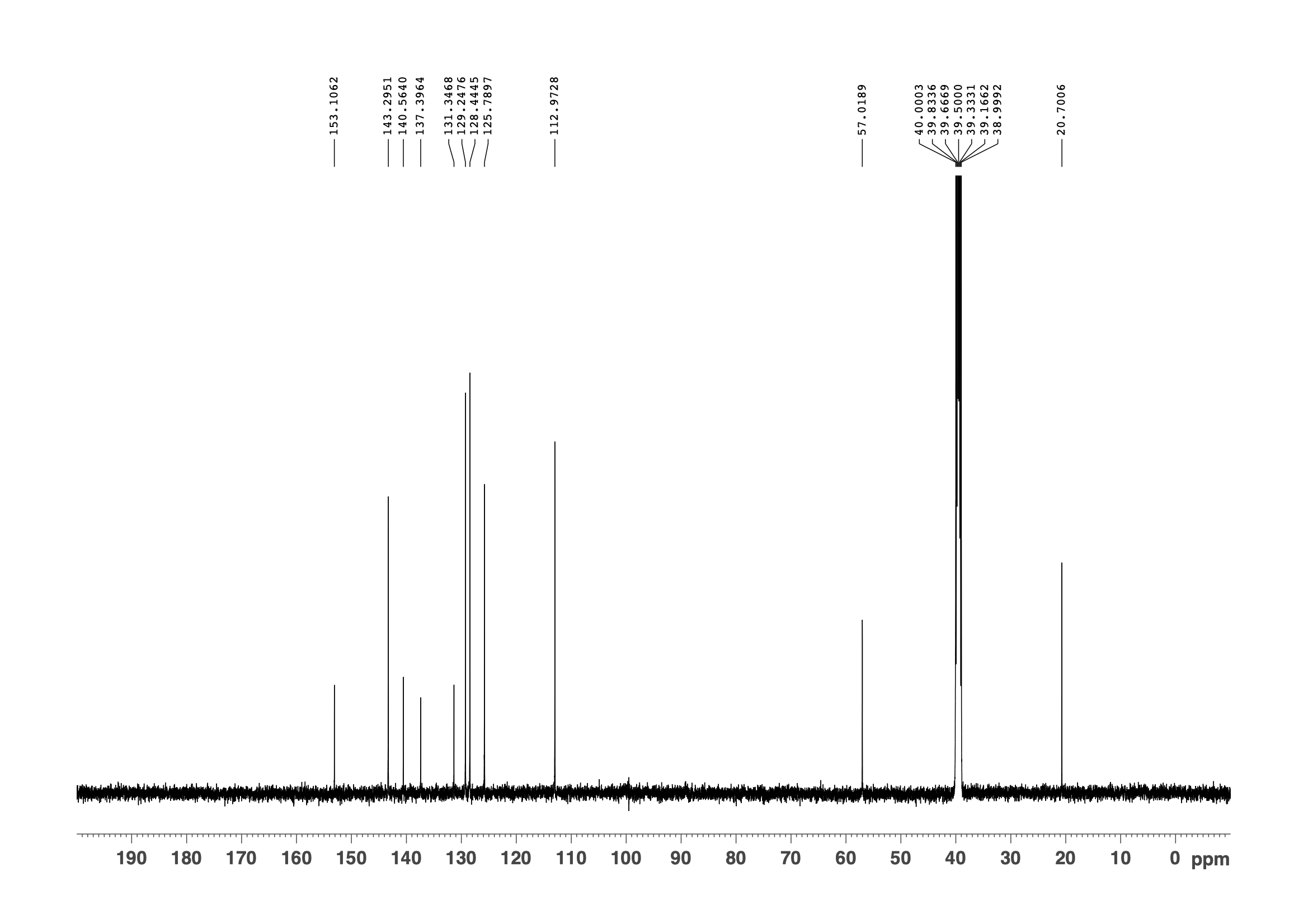


**Supplemental Fig S10.** ^1^H NMR (400 MHz) and ^13^C NMR (100 MHz) spectra of **11** (DMSO-*d*_6_).


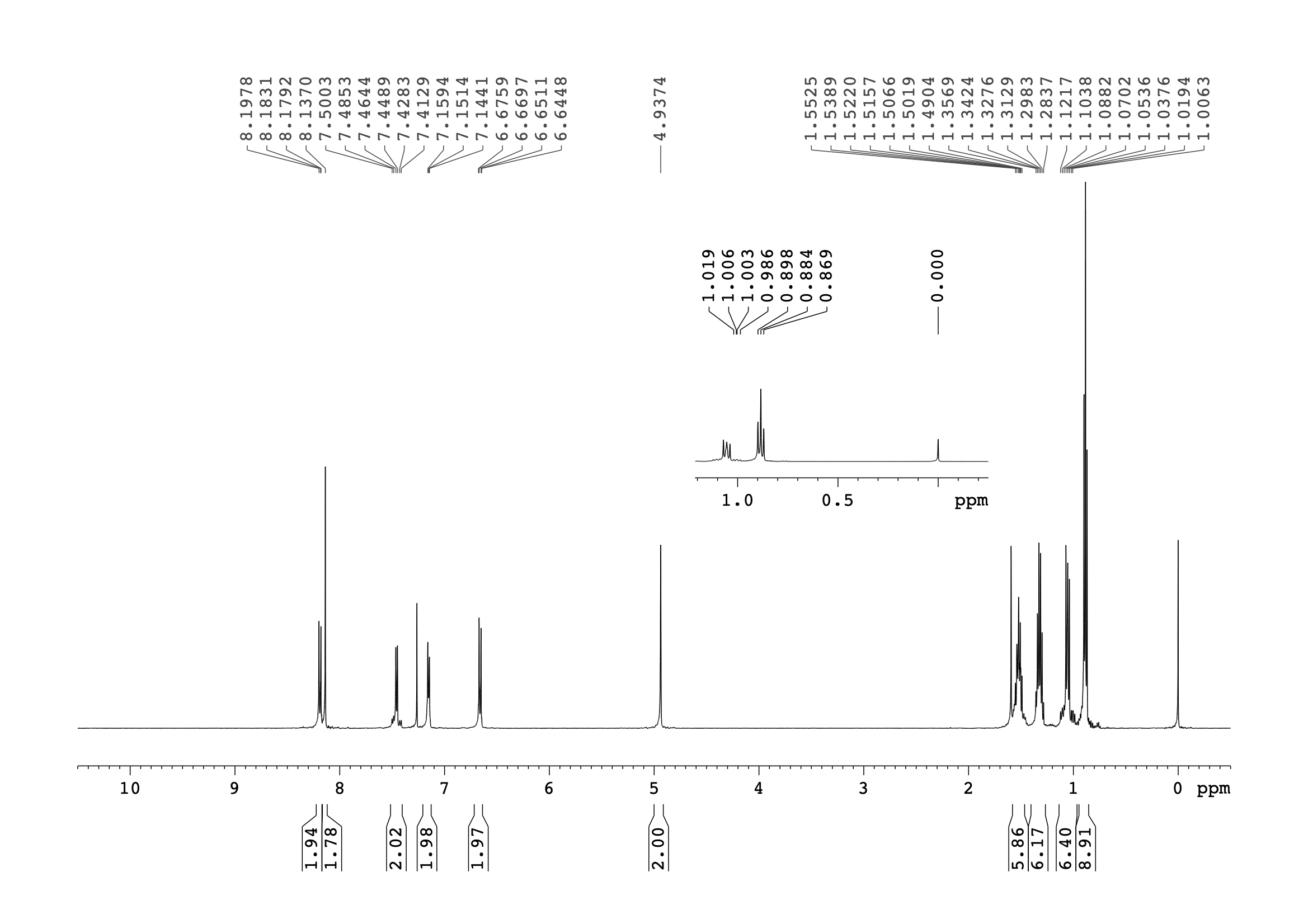


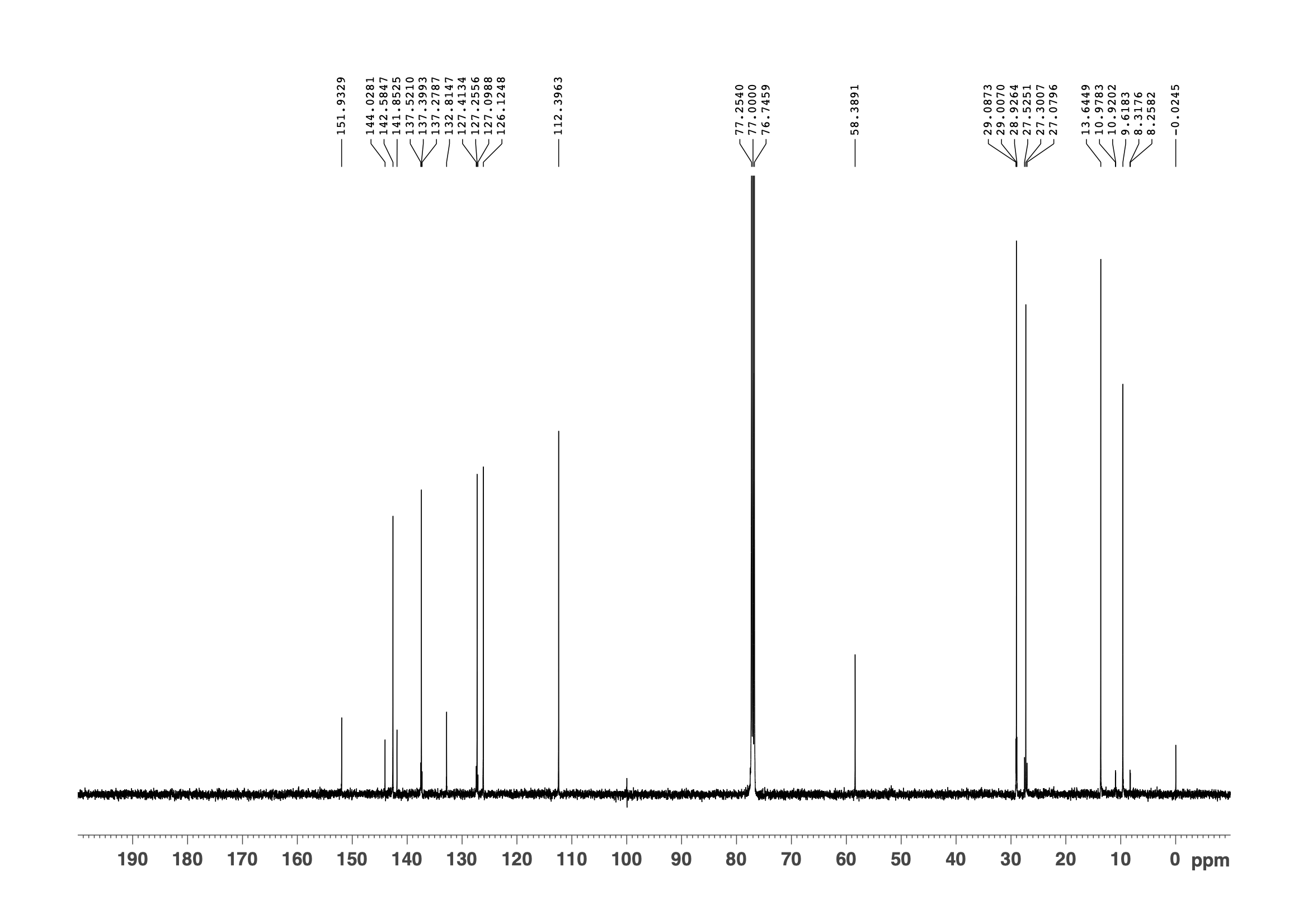


**Supplemental Fig S11.** ^1^H NMR (400 MHz) and ^13^C NMR (100 MHz) spectra of **12** (CDCl_3_).


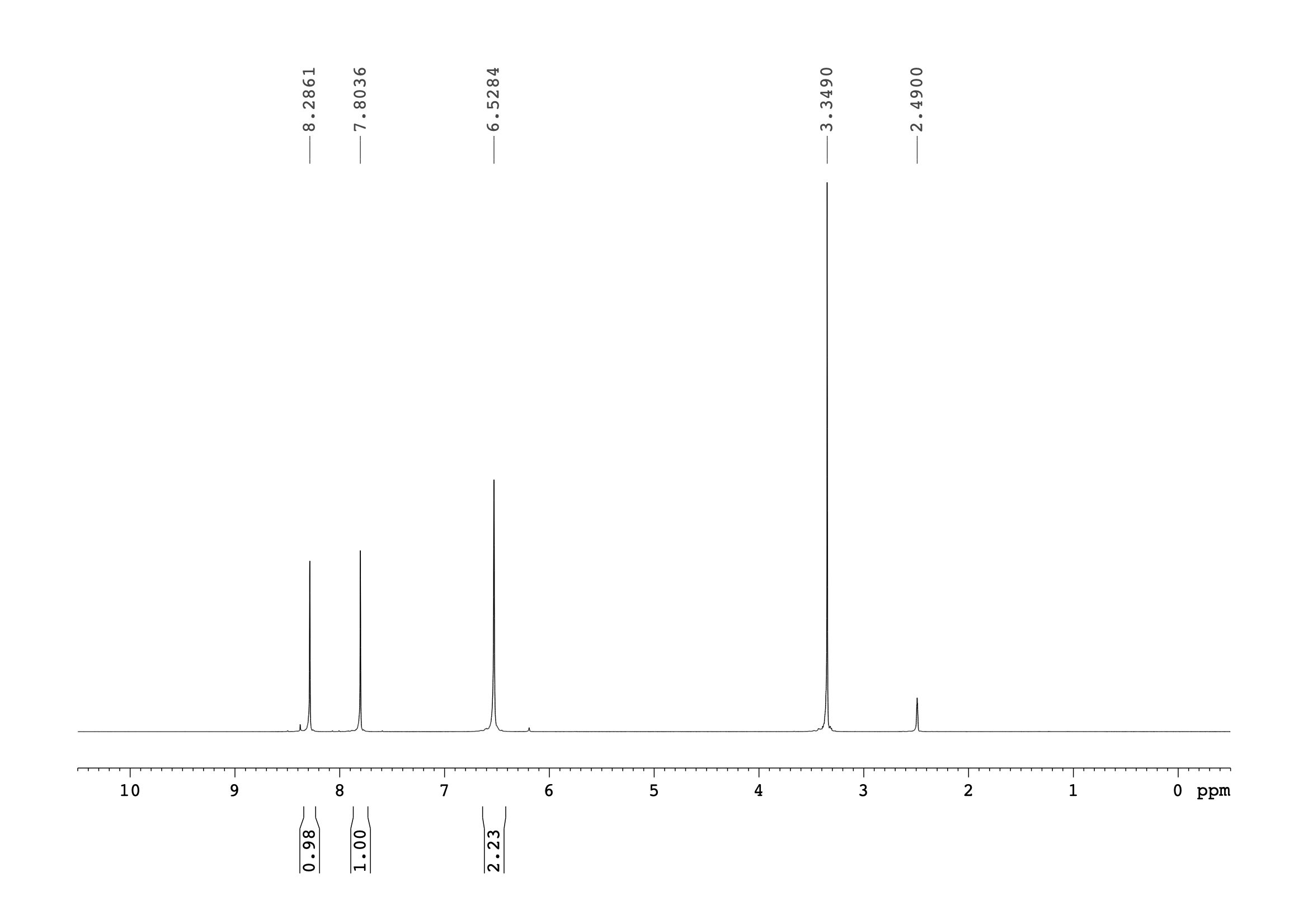


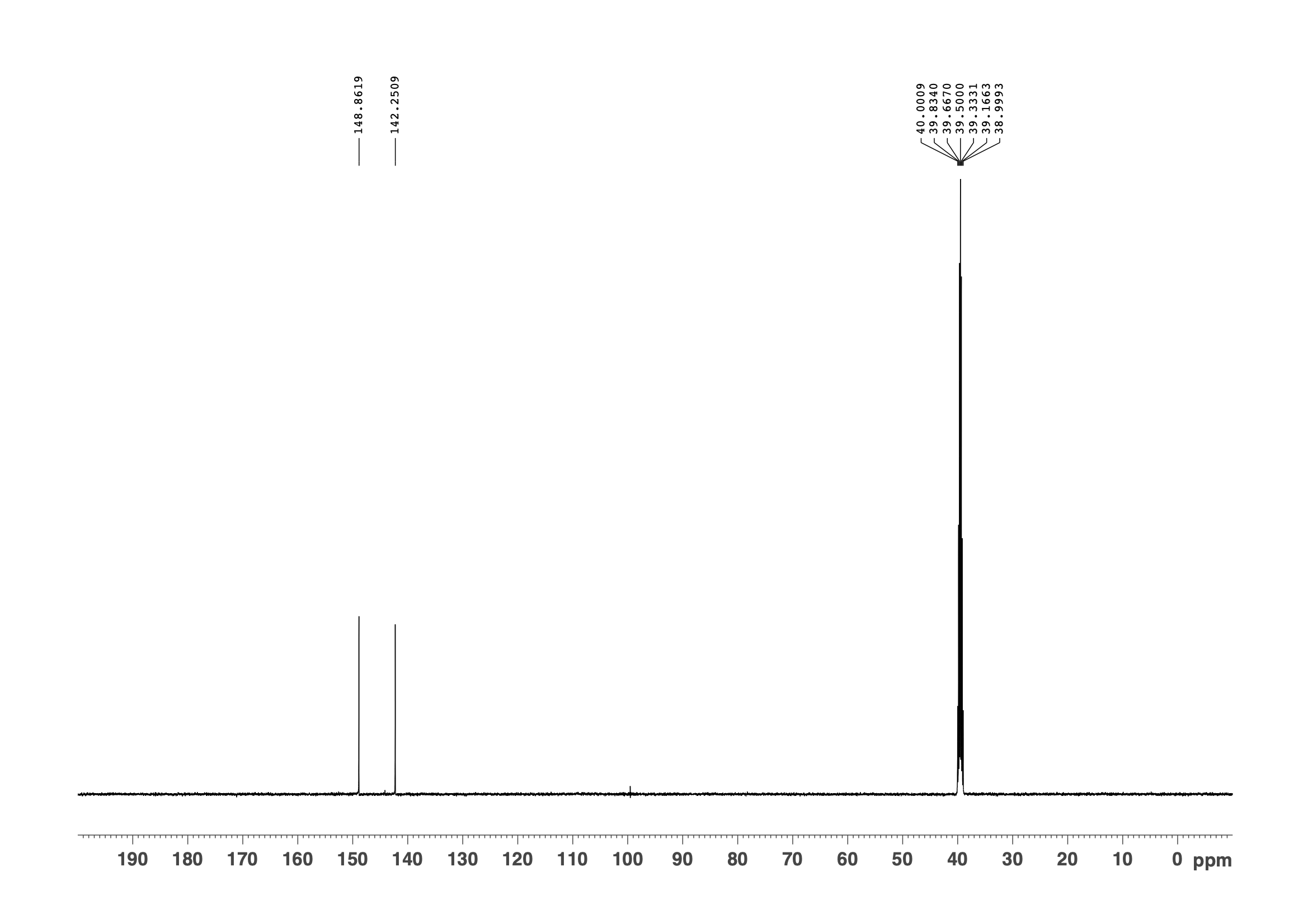


**Supplemental Fig S12.** ^1^H NMR (400 MHz) and ^13^C NMR (100 MHz) spectra of **14** (DMSO-*d*_6_).


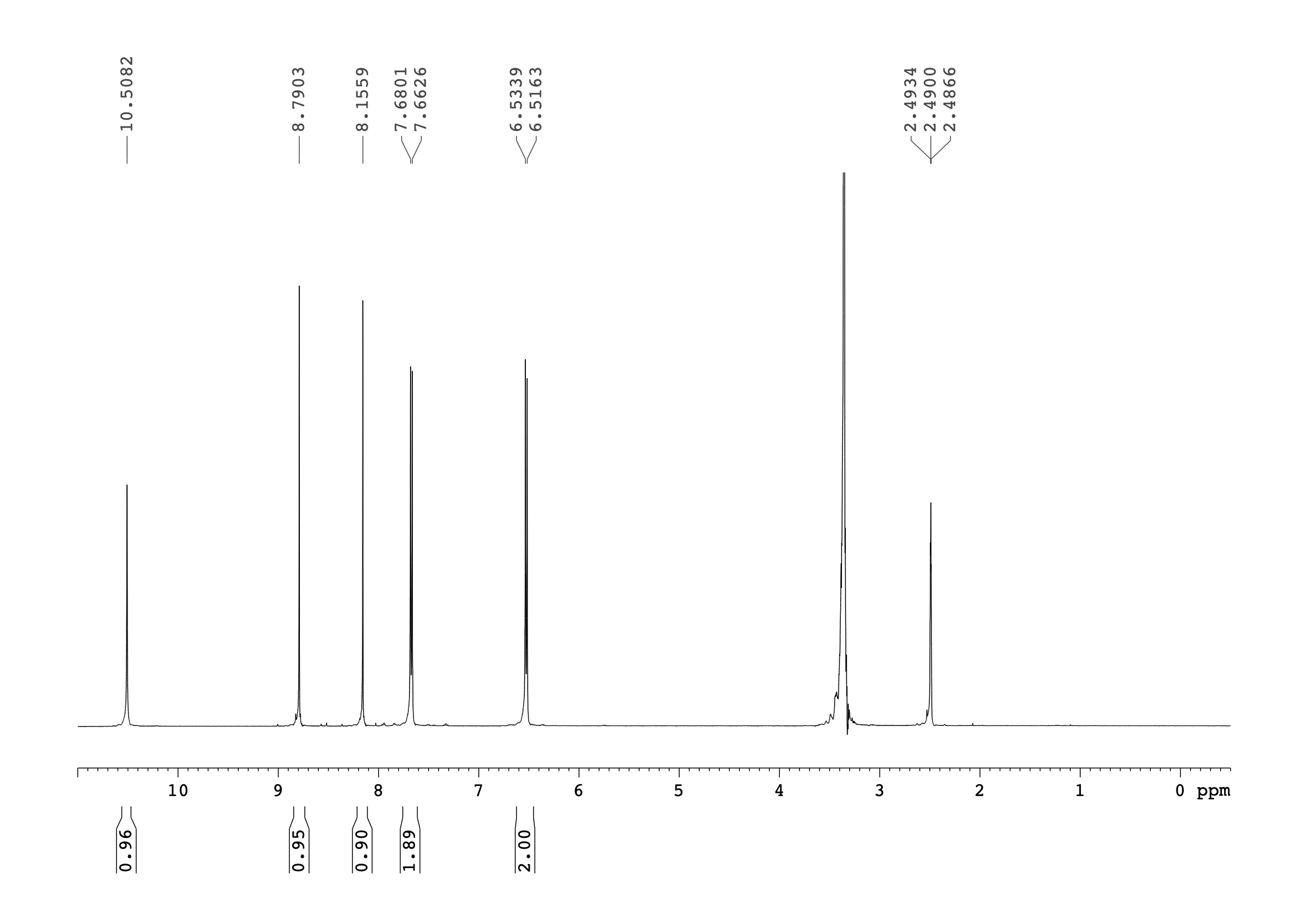


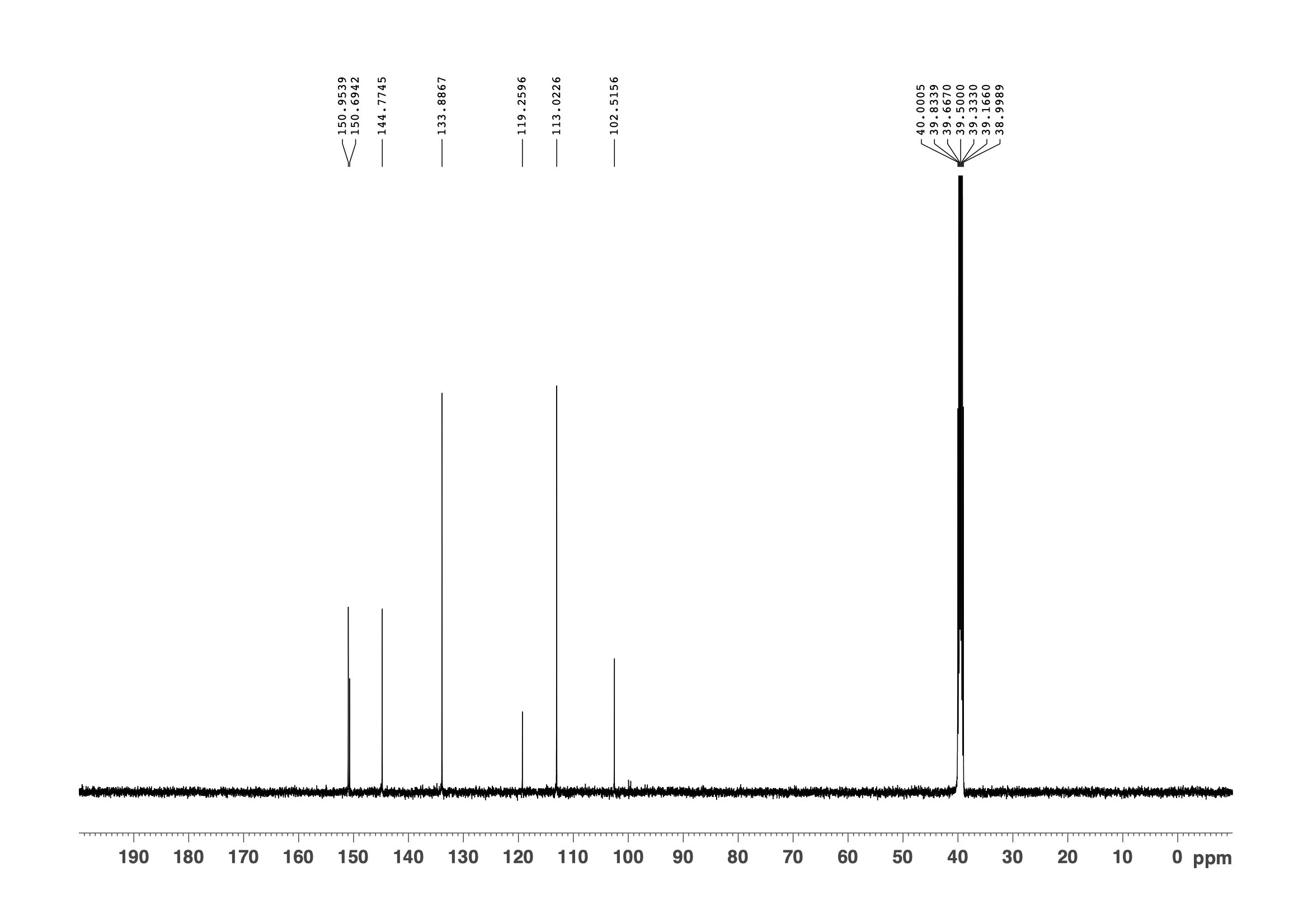


**Supplemental Fig S13.** ^1^H NMR (400 MHz) and ^13^C NMR (100 MHz) spectra of **15** (DMSO-*d*_6_).


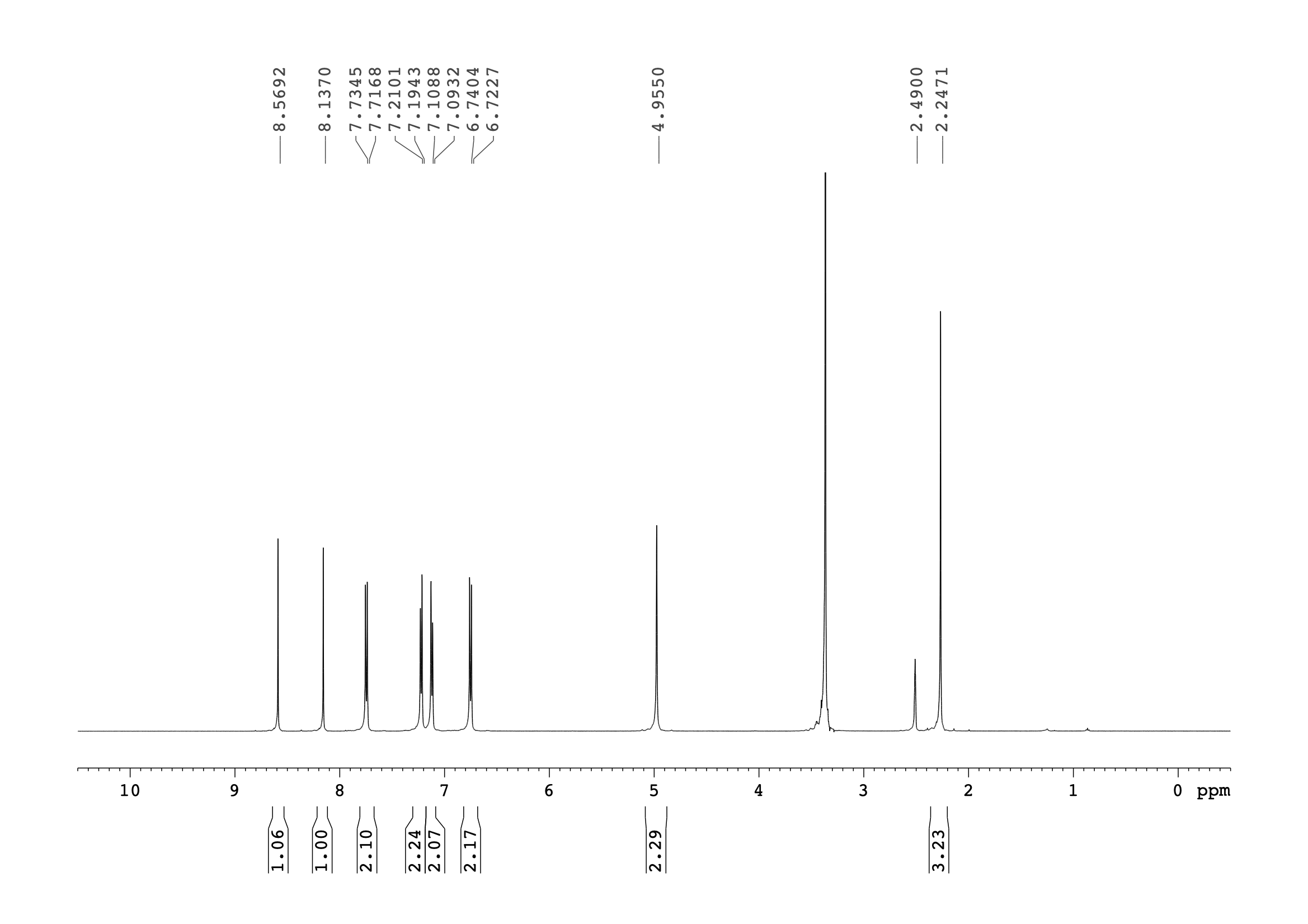


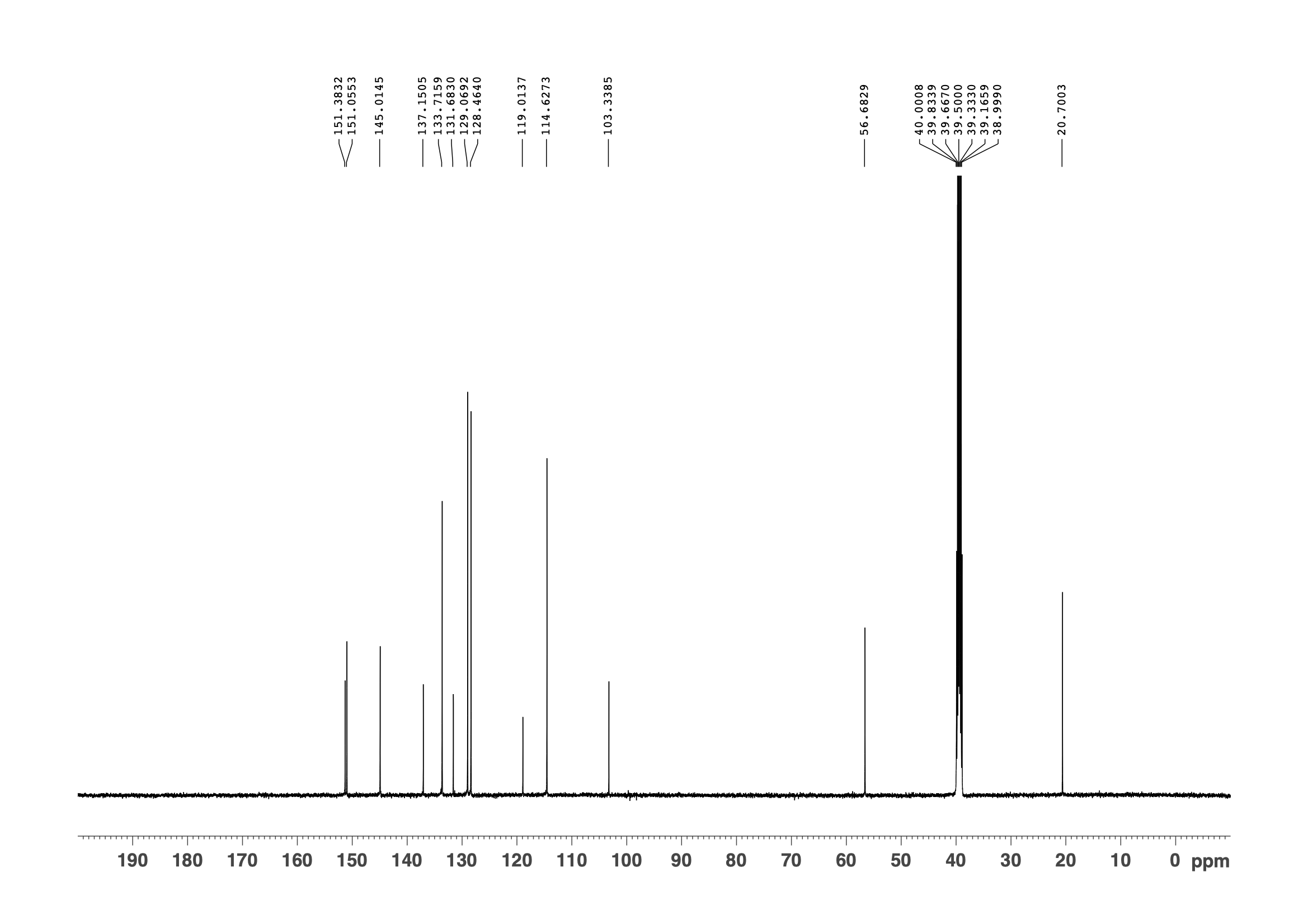


**Supplemental Fig S14.** ^1^H NMR (400 MHz) and ^13^C NMR (100 MHz) spectra of **16** (DMSO-*d*_6_).


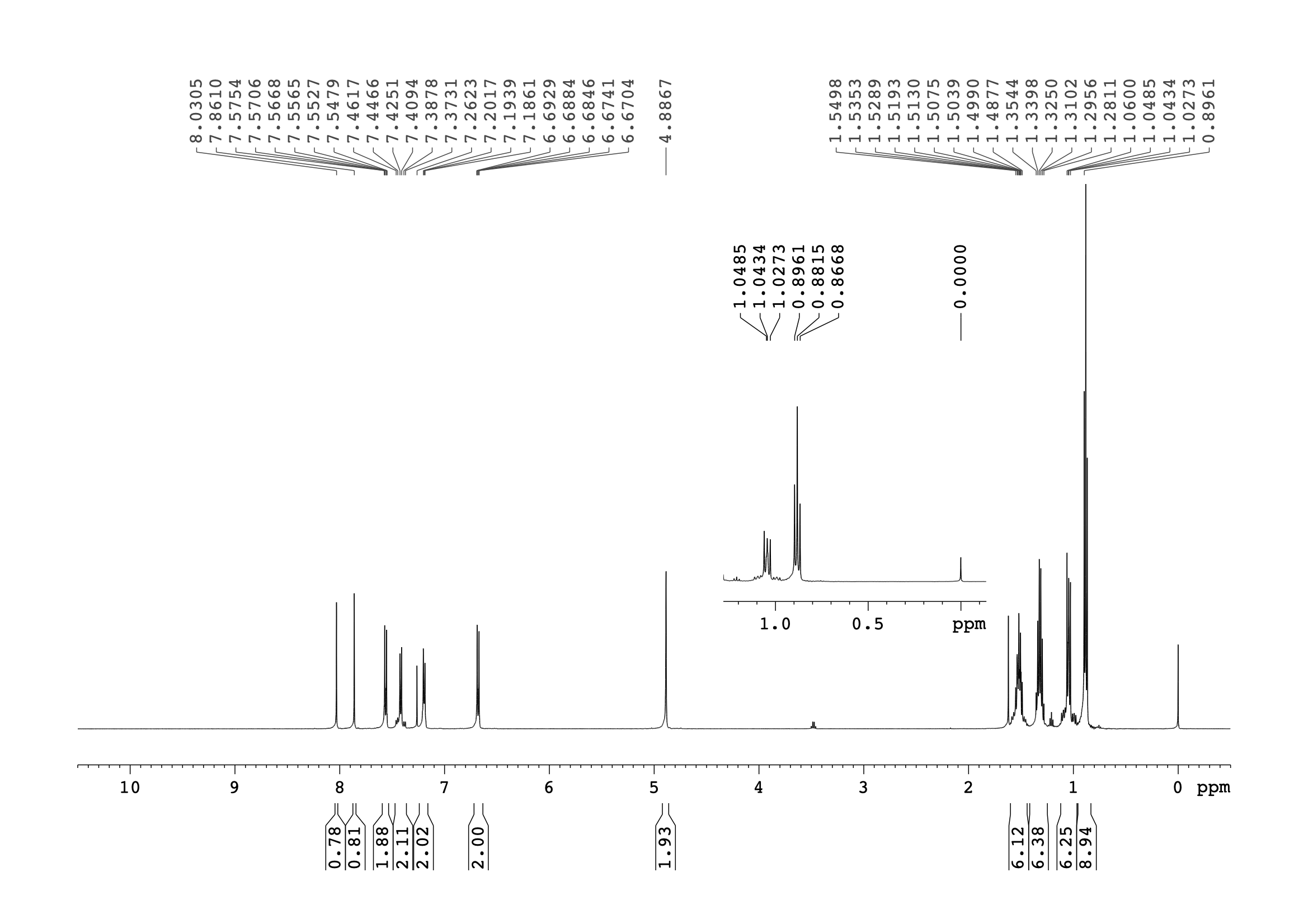


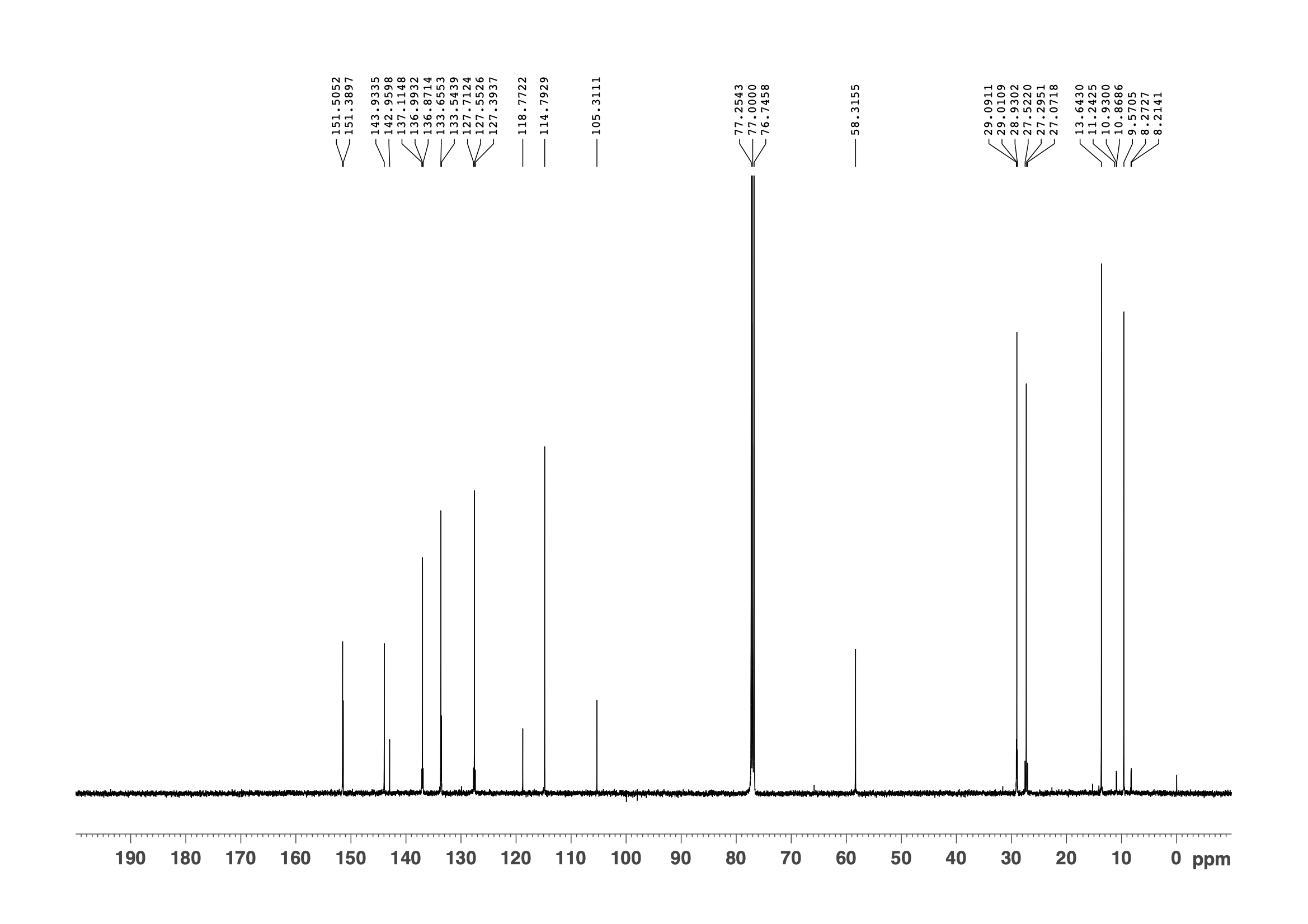


**Supplemental Fig S15.** ^1^H NMR (400 MHz) and ^13^C NMR (100 MHz) spectra of **17** (CDCl_3_).

***Radiochemistry: synthesis of ^11^C-labeled cetrozole analogs, meta-cetrozole, nitro-cetrozole, and iso-cetrozole***

[^11^C]Carbon dioxide was produced by a ^14^N(p,α)^11^C nuclear reaction using a Sumitomo CYPRIS HM-12S cyclotron (Sumitomo Heavy Industries) and then converted into [^11^C]methyl iodide by reduction of [^11^C]carbon dioxide with lithium aluminum hydride and iodination with hydroiodic acid using a RIKEN original ^11^C-radiolabeling system that carried out the heating, dilution, high-performance liquid chromatography (HPLC) injection, fractional collection, evaporation, and sterile filtration of the reaction mixture. The radioactivity was quantified using a dose calibrator. Semi-preparative purification and purity analysis using HPLC were performed on a system equipped with pumps and a UV detector, and the radioactivity of the effluent was determined using a radio analyzer.

[^11^C]Methyl iodide (approximately 30 GBq) was transferred by a stream of He gas (30 mL/min) into a solution of tris(dibenzylideneacetone)dipalladium(0) (2.7 mg, 2.4 μmol) and tri(*o*-tolyl)phosphine (3.0 mg, 5.3 μmol) in DMF (0.3 mL) in a reaction vessel (A) at room temperature. The mixture was transferred to a reaction vessel (B) containing a stannyl precursor of meta-cetrozole, nitro-cetrozole, or iso-cetrozole (**3**, **12**, or **17**; 2.5–3.0 mg, 4.4–5.3 μmol), CuCl (2.0 mg, 20 μmol), and K_2_CO_3_ (2.4 mg, 18 μmol). The inside of the reaction vessel (A) was rinsed in DMF (0.5 mL), and the solution was successively transferred to the reaction vessel (B). The resulting mixture was heated at 70 °C for 5 min. Salts and palladium residue in the reaction mixture were removed by a solid phase extraction, washing with 1 mL of a solution of CH_3_CN:30 mM CH_3_COONH_4_ (40:60, 45:55, or 50:50). The combined elutes were injected into preparative HPLC with a γ-detector. The fraction of interest was collected in a flask and concentrated in vacuo. In accordance with the radiopharmaceutical formulation, including filtration to scavenge the Pd and Cu impurities and sterilization. The desired ^11^C-labeled compound was dissolved in a mixture of polysorbate 80 (0.05 mL), propylene glycol (0.5 mL), and saline (5 mL). The pH of the solution was approximately 7 (as measured by pH paper) and the final sample volume was approximately 3.5 mL. The radioactivity of the solution was 1.2–2.6 GBq. The total synthesis time, starting from the trapping of [^11^C]CO_2_ for preparation of [^11^C]CH_3_I, was 35–36 min. The molar activities were 30–134, 44–81, and 42–170 GBq/μmol for [^11^C]meta-cetrozole, [^11^C]nitro-cetrozole, and [^11^C]iso-cetrozole, respectively. The radiochemical purity of all radiotracers was greater than 99%. The chemical purity of all radiotracers was regularly greater than 95%; however, some chemical experiments yielded 80% purity once in the synthesis of [^11^C]meta-cetrozole and 84%–88% purities twice in the synthesis of [^11^C]iso-cetrozole.

*Chromatograms and conditions of semi-preparative HPLC for isolation and analytical HPLC for purity confirmation of the synthesized [^11^C]meta-cetrozole*

Semi-preparative HPLC

Column: Cholester 10 × 250 mm; guard column: AR-II 10 × 50 mm; mobile phase: CH_3_CN containing 5% H_2_O:30 mM CH_3_COONH_4_ containing 5% CH_3_CN (40:60); flow rate: 5.0 mL/min; UV detection: 254 nm; retention time of [^11^C]meta-cetrozole: 9.8–10.9 min.

**Supplemental Fig S16.** Chromatogram of semi-preparative HPLC for isolation of [^11^C]meta-cetrozole.

Analytical HPLC

Column: AR-II 4.6 × 100 mm; mobile phase: CH_3_CN:30 mM CH_3_COONH_4_ (40:60); flow rate: 1.0 mL/min; UV detection: 254 nm; retention time of [^11^C]meta-cetrozole: 4.5 min.

**Supplemental Fig S17.** Chromatograms of analytical HPLC for purity confirmation of [^11^C]meta-cetrozole.

*Chromatograms and conditions of semi-preparative HPLC for isolation and analytical HPLC for purity confirmation of the synthesized [^11^C]nitro-cetrozole*

Semi-preparative HPLC

Column: Cholester 10 × 250 mm; guard column: AR-II 10 × 50 mm; mobile phase: CH_3_CN containing 5% H_2_O:30 mM CH_3_COONH_4_ containing 5% CH_3_CN (40:60); flow rate: 6.0 mL/min; UV detection: 225 nm; retention time of [^11^C]nitro-cetrozole: 11.5–12.5 min.

**Supplemental Fig S18.** Chromatogram of semi-preparative HPLC for isolation of [^11^C]nitro-cetrozole.

Analytical HPLC

Column: AR-II 4.6 × 100 mm; mobile phase: CH_3_CN:30 mM CH_3_COONH_4_ (40:60); flow rate: 1.0 mL/min; UV detection: 254 nm; retention time of [^11^C]nitro-cetrozole: 5.9–6.0 min.

**Supplemental Fig S19.** Chromatograms of analytical HPLC for purity confirmation of [^11^C]nitro-cetrozole.

*Chromatograms and conditions of semi-preparative HPLC for isolation and analytical HPLC for purity confirmation of the synthesized [^11^C]iso-cetrozole*

Semi-preparative HPLC

Column: Cholester 10 × 250 mm; guard column: AR-II 10 × 50 mm; mobile phase: CH_3_CN containing 5% H_2_O:30 mM CH_3_COONH_4_ containing 5% CH_3_CN (50:50); flow rate: 6.0 mL/min; UV detection: 254 nm; retention time of [^11^C]iso-cetrozole: 12.0–13.0 min.

**Supplemental Fig S20.** Chromatogram of semi-preparative HPLC for isolation of [^11^C]iso-cetrozole.

Analytical HPLC

Column: AR-II 4.6 × 100 mm; mobile phase: CH_3_CN:30 mM CH_3_COONH_4_ (50:50); flow rate: 1.0 mL/min; UV detection: 254 nm; retention time of [^11^C]iso-cetrozole: 4.3 min.

**Supplemental Fig S21.** Chromatograms of analytical HPLC for purity confirmation of [^11^C]iso-cetrozole.

***Aromatase inhibitory activity***

**Supplemental Fig S22.** Inhibitory activity of unlabeled meta-cetrozole, nitro-cetrozole, and iso-cetrozole to aromatase in the marmoset placenta. IC_50_ values were 3.50, 0.73, and 0.68, and 0.98 nM for meta-cetrozole, nitro-cetrozole, and iso-cetrozole, and cetrozole, respectively.

***Whole-body dosimetry***

Dynamic and whole-body scans were performed using an Eminence B/L PET scanner (Shimadzu, Kyoto, Japan). Six sequential whole-body scans with continuous bed motion were acquired. For the dosimetry assessment, volumes of interest were delineated on brain, liver, kidneys, urinary bladder, and gallbladder. PET images were converted to a standardized uptake value normalized to body weight and injected dose. Using the OLINDA/EXM software package, the absorbed dose was estimated. Effective dose was 2.46E-03 ± 1.70E-04 (mean ± SD, N = 2, female).

**References**

1. Okada, M.; Yoden, T.; Kawaminami, E.; Shimada, Y.; Kudoh, M.; Isomura, Y.; Shikama, H.; Fujikura, T. Studies on Aromatase Inhibitors. I. Synthesis and Biological Evaluation of 4-Amino-4*H*-1,2,4-triazole Derivatives. *Chem. Pharm. Bull.* **1996**, *44*, 1871–1879.
2. Takahashi, K.; Hosoya, T.; Onoe, K.; Doi, H.; Nagata, H.; Hiramatsu, T.; Li, X.-L.; Watanabe, Yu.; Wada, Y.; Takashima, T.; Suzuki, M.; Onoe, H.; Watanabe, Y. [^11^C]Cetrozole: an Improved C-[^11^C]Methylated PET Probe for Aromatase Imaging in the Brain. *J. Nucl. Med.* **2014**, *55*, 852–857 (2014).
3. Efange, S. M. N.; Michelson, R. H.; Khare, A. B.; Thomas, J. R. Synthesis and Tissue Distribution of (*m*-[^125^I]Iodobenzyl)trozamicol ([^125^I]MIBT): Potential Radioligand for Mapping Central Cholinergic Innervation. *J. Med. Chem.* **1993**, *36*, 1754–1760.
4. Okada, M.; Yoden, T.; Kawaminami, E.; Shimada, Y.; Kudoh, M.; Isomura, Y. Studies on Aromatase Inhibitors. II. Synthesis and Biological Evaluation of 1-Amino-1*H*-1,2,4-triazole Derivatives. *Chem. Pharm. Bull.* **1997**, *45*, 333–337.
5. (a) Jang, M. C.; Choong, P. S.; Goo, K. M.; Han, O. S.; Soo, Y. S.; Jun, P. N.; Key, C. Y.; Je, S. M. Novel 3-Substituted Cephem Compounds and Processes for Preparation Thereof. WO 1992/000981. (b) Laus, G.; Klotzer, W. Synthesis of 1-Amino-1*H*-1,2,4-triazole. *Synthesis* **1989**, 269–272.
6. Kusuhara, H.; Takashima, T.; Fujii, H.; Takashima, T.; Tanaka, M.; Ishii, A.; Tazawa, S.; Takahashi, K.; Takahashi, K.; Tokai, H.; Yano, T.; Kataoka, M.; Inano, A.; Yoshida, S.; Hosoya, T.; Sugiyama, Y.; Yamashita, S.; Hojo, T.; Watanabe, Y. Comparison of pharmacokinetics of newly discovered aromatase inhibitors by a cassette microdosing approach in healthy Japanese subjects. *Drug Metab. Pharmacok.* **2017**, *32*, 293–300.
